# Roles of Three Conserved Active Site Residues of Cytochrome *c* Nitrite Reductases During the Early Steps of Nitrite Reduction

**DOI:** 10.1021/jacs.5c03297

**Published:** 2025-07-23

**Authors:** Shahama Alam, Bradley J. Dimock, Brian Bennett, Steven J. Reinhardt, Shahid Shahid, A. Andrew Pacheco, Jarett Wilcoxen

**Affiliations:** † Department of Chemistry and Biochemistry, 14751 University of Wisconsin-Milwaukee, Milwaukee, Wisconsin 53211, United States; ‡ Department of Physics, 5505 Marquette University, 1420 W. Clybourn Street, Milwaukee, Wisconsin 53233, United States

## Abstract

Cytochrome *c* nitrite reductases (ccNiRs) catalyze reduction of nitrite to ammonium in a six-electron, eight-proton process. This report explores how the behavior of three active site ccNiR variants differs from that of the wild type enzyme (ccNiR_wt_) in the presence of nitrite and a variety of electron donors. While the nitrite-loaded active site of *Shewanella oneidensis* ccNiR_wt_ could be 2-electron reduced within seconds to yield a 6-coordinate ferrous nitrosyl ({Fe­(L)­NO}^7^) at high applied potentials, quantitative reduction of the nitrite-loaded variants R103Q, H257Q, or Y206F required substantially stronger reductants and yielded a different product over several hours. Thus, nitrite-loaded variant reduction by a large excess of hexaammineruthenium­(II) yielded quantitatively, within 2 h, a species with UV–visible spectroscopic characteristics distinct from those obtained from nitrite-loaded ccNiR_wt_ reduction. The same species was generated when the nitrite-loaded variants were treated with reduced indigo tetrasulfonate (I4S_red_), but under these conditions, the species gradually disappeared unless the nitric oxide generator 1-(*N*,*N*-diethylamino)­diazen-1-ium-1,2-diolate (DEANO) was also added to the reaction mixture. Characterization by electron paramagnetic resonance (EPR) spectroscopy of the variant species generated by I4S_red_ reduction showed them to have a 5-coordinate {FeNO}^7^ active site heme, in which the ligand L trans to the NO^•^ had dissociated from the iron center. By contrast, L did not dissociate in the 2-electron reduced wild type ccNiR. Thus, the ccNiR active site residues R103, H257, and Y206 are all needed to facilitate fast {Fe­(L)­NO}^7^ formation at high applied potentials, and to prevent dissociation of the trans ligand from {Fe­(L)­NO}^7^.

## Graphical abstract

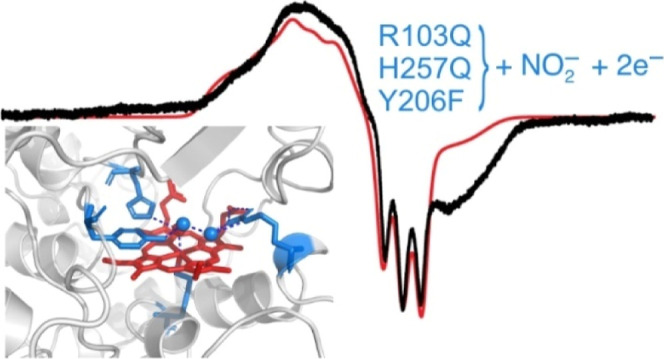

## Introduction

Cytochrome *c* nitrite reductases (ccNiRs, also called NrfAs) are found in a variety of Gram-negative bacteria.^5^ Though in many bacteria their primary role is respiratory nitrite reduction,^5,6^ ccNiRs can also reduce nitric oxide and hydroxylamine to ammonium (Scheme 1). In some microorganisms there is substantial evidence that ccNiR’s physiological role is defense against nitrosative stress,^7–12^ suggesting that nitric oxide and hydroxylamine are at least formal intermediates in the mechanism of nitrite reduction to ammonium. Under standard assay conditions that use the strong reductant methyl viologen monocation radical (MV_red_) as the electron source, no intermediate species are detected when nitrite, nitric oxide, or hydroxylamine are reduced to ammonium. However, intermediates accumulate when weaker reductants are employed, facilitating study of the ccNiR mechanism.^1,2^ Using this strategy, we examine herein important roles played by active site residues in the initial stages of nitrite reduction.

**Scheme 1:**
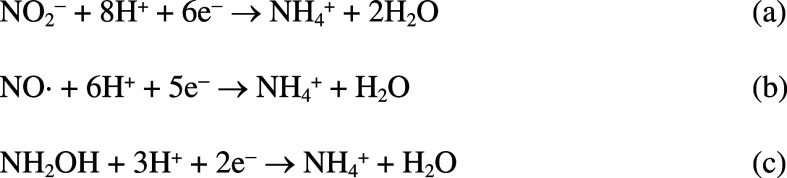
Reactions Catalyzed by ccNiR under Standard Assay Conditions^a^

a In the standard assay, the strong reductant methyl viologen monocation radical, MV_red_, is used as the electron donor.

Nitrite sits at a branchpoint in the nitrogen cycle, connecting maximally oxidized nitrate to atmospheric dinitrogen along one branch, and to maximally reduced ammonium along the other.^13–19^ Though nitrogen is incorporated into biomolecules from ammonium, nitrate provides the most abundant inorganic pool of bioavailable nitrogen. Thus, understanding the mechanisms of ammonium-nitrite interconversion is essential for understanding the nitrogen cycle. Until early in the 20th century, the only significant input of bioavailable nitrogen (“reactive nitrogen”) from the vast atmospheric dinitrogen pool was the ammonium biologically produced by nitrogen fixing microorganisms. This changed with the invention of the Haber process, which allowed ammonium to be produced industrially on a massive scale. This development was hugely beneficial in allowing humanity to feed itself, but it has come at a price. The Haber process now generates more ammonium than all natural nitrogen fixing processes and has resulted in historically high accumulation of reactive nitrogen species in the biosphere, which in turn is having serious negative consequences for the environment.^13,19–21^ The biggest problem is that ammonium fertilizer is rapidly converted to nitrate by ammonium-oxidizing bacteria, and nitrate in turn readily passes out of soil and into waterways, where it can promote the rapid growth of algae. These algae grow to such density that they consume all available oxygen, thereby killing aerobic aquatic organisms and creating massive “dead zones”, such as the one found in the Mississippi Delta, or others found in some parts of the great lakes. Nitrate itself is also toxic in high concentrations and must be removed to make water safe for human consumption. Finally, as reactive nitrogen accumulates in the environment, it is also leading to an increase in atmospheric nitrous oxide that is generated during denitrification. Nitrous oxide is not considered to be reactive nitrogen because it cannot be used as a source of bioavailable nitrogen, it can only be reduced to dinitrogen. However, because of its comparative inertness, it can persist in the atmosphere for a long time, which is a problem because it is a greenhouse gas about 300× more potent than carbon dioxide,^13^ and also an important ozone depleter.^22,23^ The study of ccNiR presented herein is part of a broader investigation aimed at better understanding the enzymatic mechanisms by which reactive nitrogen species are interconverted, in the hope that such knowledge will lead to more efficient use of ammonium fertilizer and thus lessen the problems associated with fertilizer overuse.

The typical ccNiR is a homodimer containing five *c*-hemes per protomer, with a molecular mass ranging from 52 to 70 kDa/protomer.^1,24–31^ The *Shewanella oneidensis* homologue used for the studies presented herein has a molecular mass of 52.6 kDa/protomer. Figure 1 shows the heme arrangement and active site features in the 1.66 Å resolution structure of the *S. oneidensis* ccNiR homologue (UniprotKB Q8EAC7);^1^ Figure 1a shows the relative orientations of the five hemes within one of the protomers, while Figure 1b shows key features of the active site. Four of the hemes (hemes 2–5 in Figure 1a) are low-spin and bis-his ligated, while the active site heme 1 has a high-spin ferric resting state, and, unusually, has a lysine (Lys123 in *S. oneidensis* ccNiR) bound at the proximal site instead of a histidine. The distal site of heme 1 has a loosely bound water that can be displaced by nitrite during catalysis. As with all *c*-type hemes, the ccNiR hemes are covalently bound to the protein backbone through thioether linkages to two cysteines. Hemes 2–5 interact with the protein backbone through CXXCH motifs that are characteristic of *c*-hemes;^24^ however, active site heme 1 interacts via a novel CXXCK sequence, in which K is the lysine axial ligand for the heme.^24^ The heme arrangement in ccNiR is such that the hemes are closely packed, with iron–iron distances of <13 Å, which facilitates rapid interheme electron transfer from heme 2, the likely entry point for electrons, to the heme 1 active site.^28^ Furthermore, the heme 5 groups from the two protomers are also closely spaced, which may allow interprotomer electron transfer; thus, electrons entering at heme 2 from one protomer could, in principle, end up at the active site of the other.

**Figure 1:**
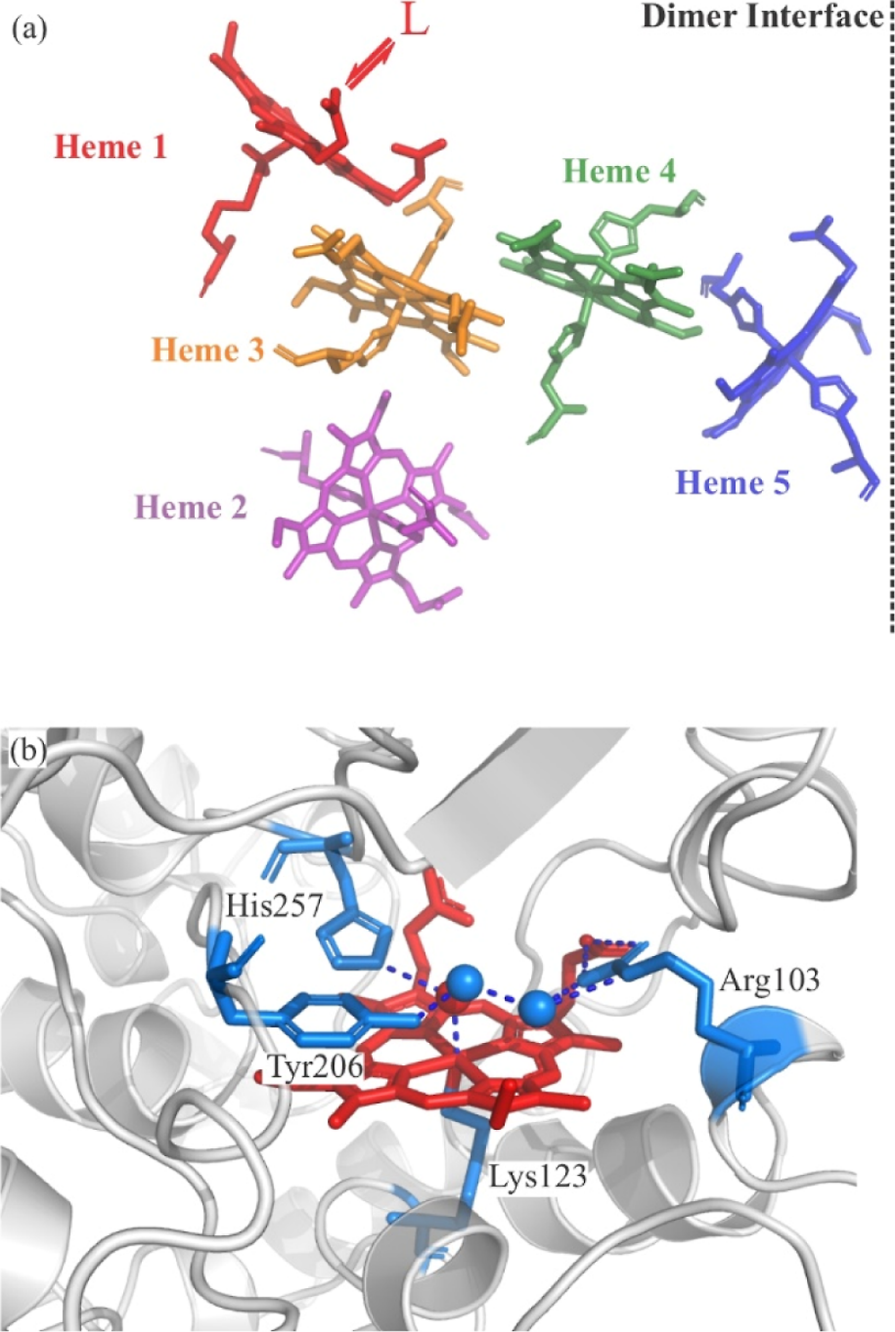
(a) Heme arrangement within one of the ccNiR protomers. Electrons are believed to enter the protein via heme 2 (purple). The four bis-His-ligated hemes conduct electrons to the heme 1 active site (red) that has the unusual lysine ligated to it. (b) *S. oneidensis* ccNiR active site showing heme 1 (red), the three conserved amino acid residues, Arg103, Tyr206, and His257 that are known to be important for catalytic activity, and the unusual proximal axial ligand, Lys123 (the key amino acid residues are shown in light blue).

All ccNiRs identified to date have three conserved amino acid active site residues that appear to be critical for optimal catalytic activity; in *S. oneidensis* ccNiR, these are Arg103, Tyr206, and His257 (Figure 1b).^1,29^ The catalytic roles of these residues have been studied in variants using a variety of methods,^32–34^ and in addition, they have been the subject of a series of detailed computational studies.^35–38^ This report focuses on the role that the three residues play in facilitating the first step in nitrite reduction at the active site and stabilizing the product of that reduction.

## Materials and Methods

### General Materials

Unless otherwise specified, reagents were obtained from commercial sources such as Fisher Scientific or Sigma-Aldrich and used without additional purification. Bovine liver catalase (Cat) was obtained from Sigma (C3155) and purified and handled as described in ref 39. The nitric oxide generator 1-(*N,N*-diethylamino)­diazen-1-ium-1,2-diolate (DEANO) was prepared using the method described by Drago and Paulik.^40,41^ Argon for inert gas manifolds and nitrogen for the gloveboxes were high purity grade and obtained from AirGas.

### General Instrumentation

Centrifugation was performed using a Sorvall Lynx 4000 and one of three rotors: the Bioflex HC, the F14-6x250y, or the F20-12x50. Routine and time-resolved ultraviolet–visible (UV–vis) spectra were recorded using one of three Cary 50 (Varian) spectrophotometers. Two of these spectrophotometers are housed in nitrogen-filled anaerobic gloveboxes for obtaining spectra of air-sensitive samples. Most UV–vis experiments were performed in 1 cm path cells, but for ccNiR samples with concentrations greater than ∼2 μM, 1 mm path cells were used. A BASi Epsilon EC potentiostat was used to electrochemically reduce indigo tetrasulfonate by two electrons and hexaammineruthenium­(III) by one. The reductions were carried out in a glovebox; a Ag/AgCl electrode was used as a reference (BASi model RE-5B) and was periodically standardized by comparison with the methyl viologen couple (ε_m_^o^ = −0.449 V vs the standard hydrogen electrode, SHE).^42^

### Active Site ccNiR Variant Expression Vector Construction and Expression

R103Q, H257Q, and Y206F variants of *S. oneidensis* ccNiR were prepared starting from the wild type expression system originally reported by Youngblut et al.^29^ This expression system consists of the small tetraheme *c* leader sequence fused to the N-terminal of the wild type ccNiR gene, inserted into a pHSG299 plasmid, and transformed into TSP-C *S. oneidensis* (an MR-1-like strain of *S. oneidensis* resistant to rifampicin).^29^ To make the desired variants separable from wild type ccNiR expressed genomically by *S. oneidensis*, the original expression system was further modified by adding to the C-terminal of the gene a TEV cleavage site, a glycine linker, a 10× histidine tag, and a two-amino-acid cap (ThrGly). The variant genes were cloned into pHSG299 (a PUC-type vector with a kanamycin resistance gene) and expressed in TSP-C *S. oneidensis* cells. Full details of the expression vector construction and transformation into *S. oneidensis* TSP-C cells are provided in Sections S1 and S2 of Supporting Information.

### Purification of ccNiR Active Site Variants from *S. oneidensis* TSP-C Cells

A detailed description of the purification procedure is provided in Section S3 of Supporting Information. Briefly, a large-scale *S. oneidensis* TSP-C bacterial culture containing the desired variant was grown in a 50 L carboy that contained 45 L LB, kanamycin (Kan, 50 μg/mL), and rifampicin (Rif, 30 μg/mL), thermostated at 30 °C in a constant temperature water bath. The culture was incubated for 16–18 h at 30 °C while being continually sparged with compressed air. The cells were harvested from the 45 L cell culture by centrifuging 1 L aliquots for 10 min at 3800*g*. The pooled cell pellets were resuspended using 20 mM tris buffer, pH = 8.1 (final volume ∼500 mL), and either PMSF (0.1–1 mM) or AEBSF (0.1–1 mM) was immediately added as a protease inhibitor. The resuspended pooled cell pellet was frozen and stored at −80 °C in a stainless-steel beaker until needed. For purification, a cell suspension containing the desired ccNiR variant was thawed, then lysed by sonication in a water/ice-cooled stainless-steel beaker. The suspension was then centrifuged at 30,000*g* for 60 min to remove cell debris, and the clarified supernatant was separated. Imidazole was dissolved into the supernatant to 40 mM, which was then filtered using a 0.2 μm syringe filter and loaded onto an affinity column (GE Healthcare HisTrap FF, 20 mL). This column was pre-equilibrated with a buffer (Buffer A) that contained 40 mM imidazole, 20 mM tris base, and 500 mM NaCl, adjusted to pH = 8.1. After the clarified cell extract had been loaded, the column was washed with 5–10 column volumes of Buffer A until the UV reading at 280 nm (A280) of the column flow-through was once again constant. The desired his-tagged variant was eluted by mixing Buffer A with 80% of a pH 7.0 buffer containing 500 mM Imidazole, 20 mM HEPES, and 500 mM NaCl. All fractions for which the A280 deviated significantly from the baseline were pooled and immediately transferred to Snake-Skin dialysis tubes (9 mL/1 cm, ThermoFisher) for roughly 4 h of dialysis at 4 °C in a low salt buffer reservoir (20 mM HEPES, 500 mM NaCl, pH = 7.0). The His-tag was removed by incubating the protein with tobacco etch virus (TEV) protease. TEV protease was added to the dialysis tube containing the desired variant according to the protocol of adding 1.0 O.D. TEV protease to 10 O.D mutant ccNiR. The dialysis tube was then transferred into another buffer reservoir (50 mM Tris, 100 mM NaCl, 1 mM DTT, 250 μM EDTA, pH = 8.0) which is an optimum buffer for TEV protease activity. The digestion of His-tag by TEV protease continued overnight at 4 °C. The digested protein solution was centrifuged at 39,000*g* for 10 min and the supernatant was collected. A HisTrap FF column (5 mL, GE Healthcare) was equilibrated with a buffer containing 25 mM tris, 300 mM NaCl, pH = 8.0, after which the clarified protein solution was loaded onto it. This column captured undigested variant ccNiR and the TEV protease, which is also His-tagged, but allowed the variant ccNiR from which the His-tag had been successfully cleaved to pass through. The buffer from the flow-through was exchanged for a buffer containing 50 mM HEPES, 150 mM NaCl, pH = 7.0, and the resulting solution was concentrated using centrifugal concentrators (Amicon) spun at 4000*g* for 10 min intervals. Size exclusion chromatography (SEC, Sephacryl-S200, GE Healthcare) was used as the final step of purification. The SEC column (320 mL) was equilibrated with the buffer containing 50 mM HEPES, 150 mM NaCl, pH = 7.0, and then loaded with a concentrated variant ccNiR sample (not more than 2.5 mL). Two distinct A280 peaks were observed in the chromatograph. The second of these typically displayed UV–vis ratios A409/A280 > 3.8 and was confirmed to be the pure variant ccNiR by SDS-PAGE. The pure protein’s buffer was exchanged for 50 mM HEPES at pH = 7.0, after which the solution was concentrated using centrifugal concentrators and stored at −80 °C.

### UV–vis Analysis of Time-Resolved Nitrite-Loaded Variant ccNiR Reduction

Stock solutions of 0.1 M sodium nitrite, 10 mM hexaammineruthenium­(III), or 600 μM indigo tetrasulfonate (I4S) were made inside a glovebox using anaerobic 50 mM pH 7 HEPES buffer. Hexaammineruthenium­(III) was fully reduced to hexaammineruthenium­(II) (Ru^II^), and I4S to its 2-electron reduced form (I4S_red_), by controlled potential electrolysis at −40 mV and −200 mV vs SHE, respectively, using the BASi Epsilon EC potentiostat. For experiments requiring partially reduced electron donors (Section S5, Supporting Information), the applied potential was adjusted accordingly. Stock 20 mM solutions of DEANO were made in the glovebox using anaerobic 1 mM NaOH (pH 11). Variant ccNiR was brought through the glovebox antechamber frozen, then made thoroughly anaerobic by repeated exchanges with 50 mM pH 7 HEPES buffer, accomplished using microcentrifugal concentrators spun at 14,000 rpm in an Eppendorf miniSpin Plus centrifuge. For UV–vis experiments with variant ccNiR concentrations less than 2 μM, the stock solutions after buffer exchange were typically ∼60 μM, while ∼400 μM stock solutions were used for the UV–vis and EPR experiments that required high variant ccNiR concentrations. Time-resolved UV–vis experiments were performed using a Cary Bio 50 spectrophotometer in multiple-scan mode. In a typical experiment with low ccNiR concentrations, an aliquot of Ru^II^ stock was added to a 1 cm semimicro cuvette already containing variant ccNiR and nitrite; the total solution volume was always 1 mL. The reagents were rapidly mixed using a cuvette mixer (Fireflysci P68 mixer for semimicro cuvettes), and data collection was immediately initiated. Spectra were collected every 15 s for 1 h in the range 300 nm −800 nm (240 scans total). Data were collected in csv format and later analyzed by using programs written for Mathcad 15.0 (PTC Software) and Origin 9.0 (Microcal Software).^43,44^ For experiments involving variant ccNiR concentrations greater than 2 μM, a slightly different procedure was used. Variant ccNiR, nitrite, and I4S_red_ were combined with anaerobic 50 mM pH 7 HEPES buffer in volumes that provided the desired reagent concentrations (see Results), but such that the total solution volume was less than 300 μL. The solution was rapidly mixed with a 1 mL Eppendorf pipettor and then transferred to a 1 mm path length quartz cuvette. The cuvette was capped to minimize potential escape of nitric oxide and placed in the Cary Bio 50 spectrophotometer, where spectra were collected every minute for 30 min in the range 300 nm–800 nm using multiple-scan mode. After 30 min, an aliquot of the DEANO stock solution was added directly to the cuvette to give a concentration of 300 μM, and additional spectra were collected in the range 300 nm–800 nm for another hour.

### EPR Analysis of the Nitrite-Loaded Variant ccNiR Reduction Product

For the R103Q variant, three EPR samples were prepared in a glovebox, all of which contained 60 μM R103Q and 2 mM NO_2_^–^, in a total volume of 200 μL. After the appropriate preparation (described next), each sample was transferred to a 4 mm OD quartz, thin wall, EPR tube (Wilmad), frozen in a dry ice–ethanol solution while still in the glovebox, sealed in a Ziplock bag, then brought out of the glovebox and immediately transferred to liquid nitrogen for storage until needed. The first sample contained no other reagents and was frozen without further manipulation; this provided the spectrum of fully oxidized nitrite-loaded R103Q ccNiR. The second sample additionally contained 120 μM I4S_red_ and was frozen after a 30 min incubation period. The final sample also initially contained 120 μM I4S_red_, but after a 30 min incubation period, 1.8 mM DEANO were also added to the solution, after which it was incubated for 60 more minutes before freezing. Samples containing 2 mM NO_2_^–^, 120 μM I4S_red_, 1.8 mM DEANO, and 60 μM of H257Q or Y206F ccNiR were also prepared as described above for R103Q ccNiR.

X-band (9 GHz) continuous wave EPR spectroscopy of the R013Q variant was initially carried out on a Bruker EMXTDU/L E4002501 spectrometer equipped with E532LX digital acquisition, an ER4116 DM resonator, a ColdEdge/Bruker ER4112HV-S5-L Stinger cryogen-free 5–100 K closed cycle helium cryocooler, an HP 5350B microwave counter, and an Oxford Instruments ESR900 cryostat with Mercury-ITC temperature controller. Spectra were typically recorded at approximately 9.644 (±0.005) GHz microwave frequency, 2.6 mW microwave power, 10 G (1.0 mT) magnetic field modulation amplitude at 100 kHz, 1.2 G (0.12 mT) digital field resolution, and a temperature of 10 K. Other acquisition parameters were chosen such that the spectral resolution was limited by the modulation amplitude. Background signals were recorded on a frozen water sample and subtracted using the Bruker Xenon software. Following the initial spectroscopic characterization of R103Q, EPR spectra were obtained at 100 K for all three variants, R103Q, Y206F and H257Q, using a Magnettech MS5000 spectrometer (Freiberg Instruments) equipped with a TCH04 liquid nitrogen cryostat, which includes a temperature and gas-flow controller. Samples were measured under nonsaturating conditions at approximately 9.475 (±0.005) GHz microwave frequency, 10 mW microwave power, 60 s sweep time, and 5G (0.5 mT) modulation amplitude. The EPR signals of ccNiR variants were quantified using 100 μM and 200 μM Cu-EDTA spin standards. The spectra were corrected for temperature, power, modulation amplitude, and quality factor before double integration and compared to the standard to yield a spin concentration for each sample.^45^ Spectral analysis, plotting, and simulations were performed using Easyspin^46^ (version 6.0.6, Easyspin.org) in Matlab R2024b (Mathworks Inc.). All spectra, collected on either Bruker or Magnettech spectrometers, are shifted from their collection frequencies, which vary from 9.472 to 9.649 GHz, to a common 9.475 GHz for plotting and spectral comparison by using eq 1 below.

1 $$x\left(data@9.475\;GHz\right))=x\left(data@orig\;freq\right)\times \frac{9.475\;GHz}{v\left(of\;collected\;data,GHz\right)}$$

## Results

### Overview

In two previous reports we showed that exposing nitrite-loaded wild type ccNiR (ccNiR_wt_) to very weak reductants, such as *N*,*N*,*N′*,*N′*- tetramethyl-*p*-phenylenediamine (TMPD), results in a 2-step reduction of the ccNiR active site, as shown in Scheme 2.^1,2^ In the first reduction step, which has a calculated midpoint potential of 0.13 V vs SHE, the nitrite loaded active site, [Fe^III^_H1_(NO_2_^–^)], is reduced to [Fe^II^_H1_(NO_2_^–^)] (step 1, Scheme 2). The second step couples nitrite dehydration with a second 1-electron reduction to form a ferrous nitrosyl species referred to as {Fe_H1_NO}^7^ using the Enemark–Feltham notation (step 2, Scheme 2).^3^ This step has an estimated midpoint potential of 0.37 V vs SHE, which is much higher than the midpoint potential of the first step, making the intermediate [Fe^II^_H1_(NO_2_^–^)] unstable with respect to disproportionation. Indeed, [Fe^II^_H1_(NO_2_^–^)] was only transiently observed in stopped-flow experiments when nitrite-loaded ccNiR_wt_ was reduced with TMPD.^2^ On the other hand, the 2-electron reduced {Fe_H1_NO}^7^ moiety was indefinitely stable in the absence of stronger reducing agents (but see proviso below, regarding ccNiR-catalyzed reduction of nitrite to NO^•^). This is because the net midpoint potential for the 2-electron reduction of [Fe^III^_H1_(NO_2_^–^)] to {Fe_H1_NO}^7^ was estimated to be 0.25 V vs SHE (step 3, Scheme 2), which is about 0.3 V above the potential needed to effect significant conversion of nitrite to ammonium.^1,2^ Thus, once formed, the {Fe_H1_NO}^7^ moiety could be reduced no further by TMPD.

**Scheme 2:**
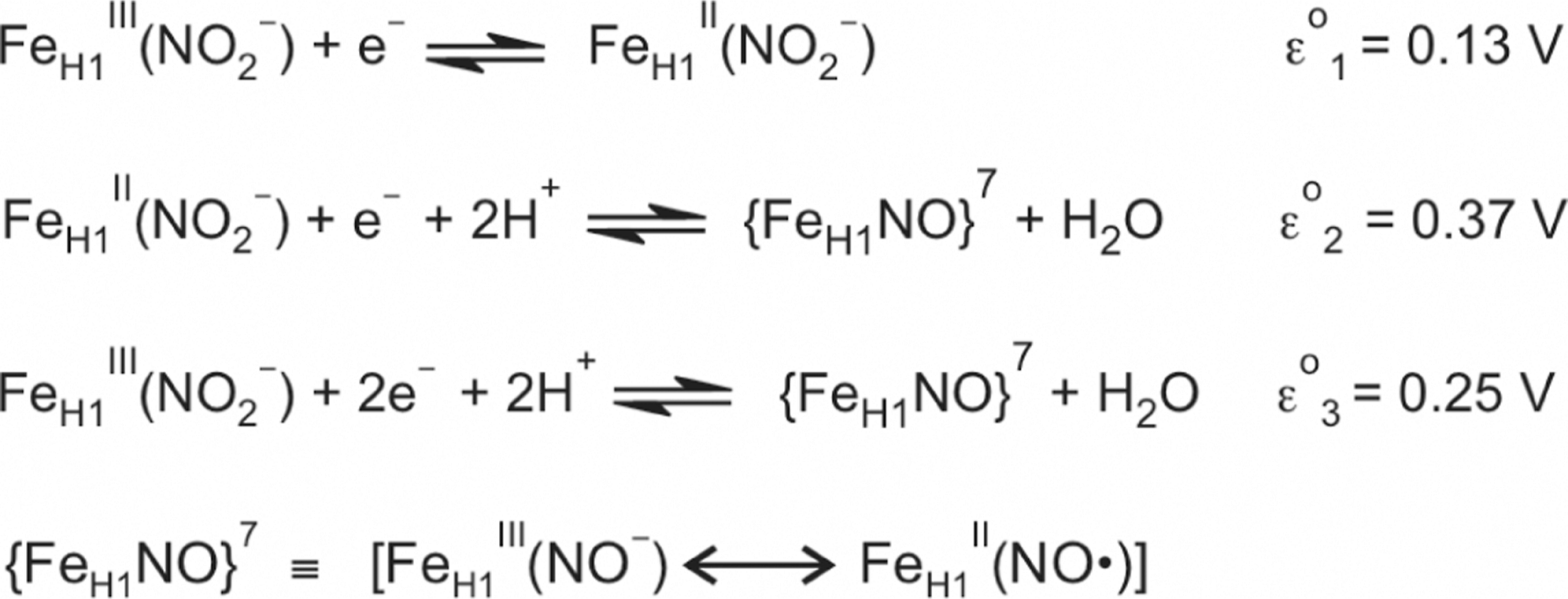
Steps by Which Nitrite-Loaded ccNiR_wt_ Is Reduced in the Presence of Weak Reductants, Such as TMPD^1,2^ ^a^

a {Fe_H1_NO}^7^ is the product of 2-electron reduction, represented using the Enemark–Feltham notation,^3^ defined at the bottom of the scheme.

Preliminary experiments in which the nitrite-loaded ccNiR variants H257Q, R103Q, and Y206F, were exposed to TMPD and similarly weak reductants showed that these variants did not undergo the same 2-electron reduction to {Fe_H1_NO}^7^ as ccNiR_wt_, though some very slow spectral changes were still observed by UV–vis spectroscopy.^4,47^ The experiments that follow used the slightly stronger electron donors hexaammineruthenium­(II) and reduced indigo tetrasulfonate to effect the same spectral changes at more convenient rates.

### Reactivity of Nitrite-Loaded R103Q ccNiR with Pure Hexaammineruthenium­(II)

Figure 2a shows the spectral changes observed after mixing 1.15 μM R103Q ccNiR with 4.7 mM nitrite and 1.0 mM Ru^II^, in a pH 7 HEPES buffer. Singular value decomposition (SVD) analysis revealed that three spectral components are needed to faithfully reconstruct a noise-reduced absorbance matrix. The SVD-treated data were then fit with eq 2a using a global fitting routine (red traces, Figure 2a).^43,44^

2a $$A_{\lambda ,t}=S_{0\left(\lambda \right)}+S_{1\left(\lambda \right)}f_{1}\left(t\right)+S_{2\left(\lambda \right)}f_{2}\left(t\right)$$

2b $$f_{1}\left(t\right)=\frac{k_{app1}}{k_{app2}-k_{app1}}\left[\exp\left(-k_{app1}t\right)-\exp\left(-k_{app2}t\right)\right]$$

2c $$f_{2}\left(t\right)=\frac{1}{k_{app2}-k_{app1}}\left\{k_{app2}\left[1-\exp\left(-k_{app1}t\right)\right]-k_{app1}\left[1-\exp\left(-k_{app2}t\right)\right]\right\}$$

In eq 2a, *A*_λ*,t*_ is the absorbance obtained at wavelength λ and time *t*, and **S**_**0**_–**S**_**2**_ are the spectral components. Component **S**_**0**_ is present at *t* = 0, **S**_**1**_ grows exponentially at a rate governed by *k*_app1_ and then decays exponentially at a rate governed by *k*_app2_ (eq 2b), and **S**_**2**_ grows in as **S**_**1**_ decays, at a rate governed by both *k*_app1_ and *k*_app2_ (eq 2c). Note that **S**_**0**_ should decay exponentially as **S**_**1**_ grows in, at a rate governed by *k*_app1_. When **S**_**0**_ is treated as a constant, **S**_**1**_ and **S**_**2**_ will appear as difference spectra, which is helpful when the spectral change is small compared to the absolute spectral intensity. Figure 2b shows an absorbance vs time slice from the Figure 2a data set,obtained at 409 nm, which is the Soret maximum for fully oxidized R103Q ccNiR. The blue trace is experimentally obtained, the red one is from the least-squares best fit with eq 2a. The fit is excellent, showing that eq 2a provides a good mathematical description of the experimentally observed spectral changes. Note though, that eq 2a is purely empirical; we ascribe no mechanistic significance to it.

**Figure 2:**
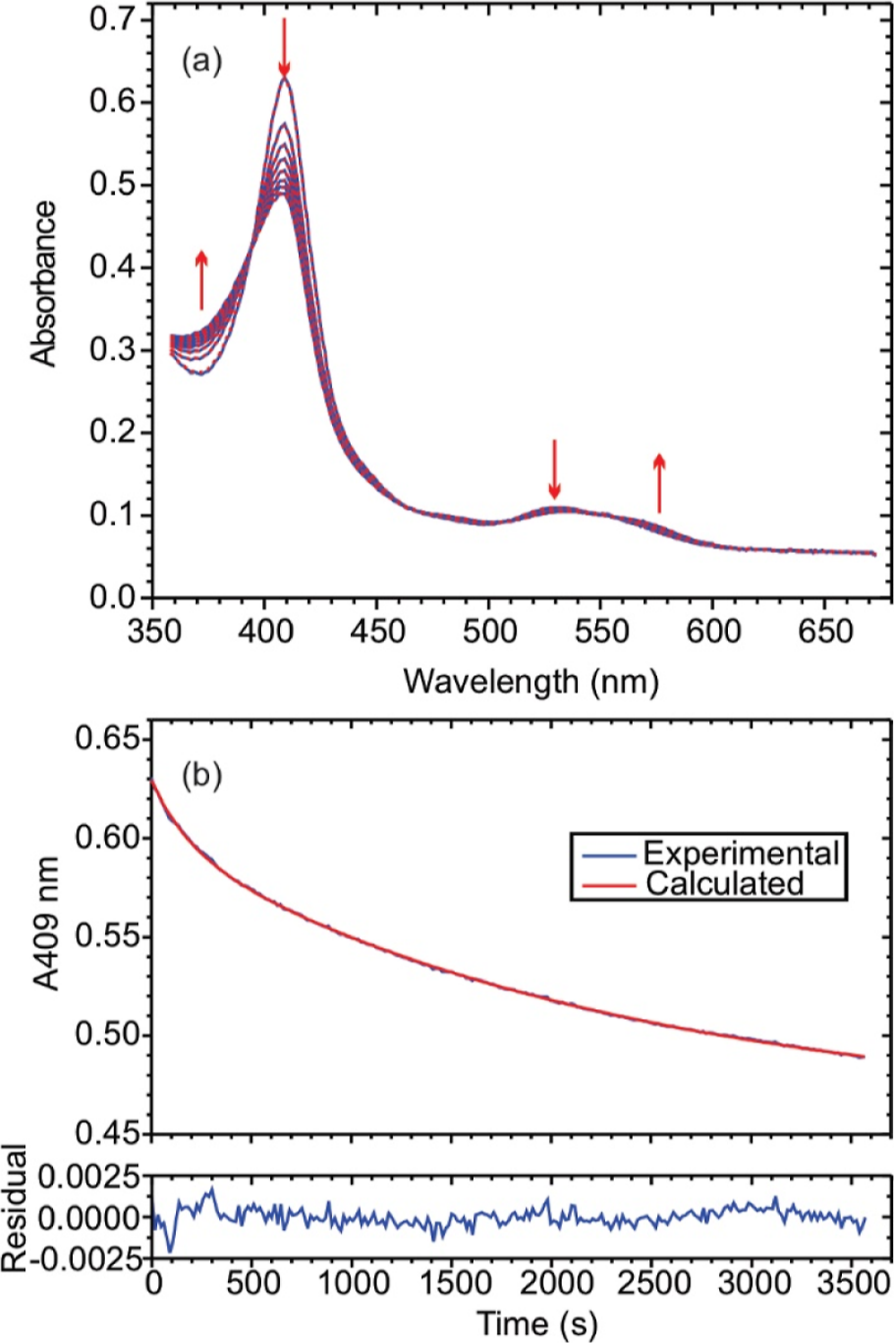
(a) Blue traces: UV–vis spectral changes observed after mixing 1.15 μM R103Q ccNiR with 4.7 mM nitrite and 1.0 mM Ru^II^, in a pH 7 HEPES buffer. Dashed red traces: least-squares best fit with eq 2a. The reaction mixture was monitored at 15 s intervals, but for clarity, the selected traces are at 600 s intervals. (b) Blue trace: absorbance vs time slice taken at the 409 nm maximum of the data set above. Red trace: least-squares best fit with eq 2a.

The blue trace in Figure 3a shows the spectral component **S**_**0**_ that was obtained by fitting the Figure 2a data with eq 2a. The component **S**_**0**_ was in turn fit with the independently obtained extinction coefficient spectra of fully oxidized R103Q and nitrite; nitrite absorbs only slightly, and 1 mM Ru^II^ has negligible absorbance, in this wavelength range. Notice that the best fit of **S**_**0**_ in Figure 3a with the oxidized R103Q extinction coefficient spectrum leaves a substantial residual spectrum (Figure 3b), with absorbance increases at 423 and 551 nm, and a decrease at 407 nm, a pattern that is characteristic of low-spin bis-his ligated *c*-heme reduction.^48,49^ In our earlier studies of ccNiR_wt_ reduction by weak reductants in the presence of nitrite,^1,2^ we found that the difference spectra obtained upon heme 1 active site reduction lacked the 551 nm peak but did exhibit an absorbance increase at 423 nm and decrease at 402 nm, together with a broad increase centered at ∼555 nm. The presence of the sharp 551 nm peak in the Figure 3b residual therefore suggests that one of the bis-His ligated low-spin hemes (Figure 1a) was at least partially reduced within the mixing time. The highest-potential heme after heme 1 in ccNiR_wt_ is heme 4 (Figure 1a),^50^ so we tentatively ascribe the appearance of the 551 nm peak to reduction of heme 4 that takes place within the mixing time when 1 mM Ru^II^ is the electron donor. Note that under more weakly reducing conditions, such as when 1:1 Ru^II^/Ru^III^ or TMPD is the electron source, the **S**_**0**_ component is well fit by the oxidized R103Q extinction coefficient spectrum (Section S5, Supporting Information). This shows that, under the less strongly reducing conditions, R103Q remains fully oxidized during the mixing time of the experiment.

**Figure 3:**
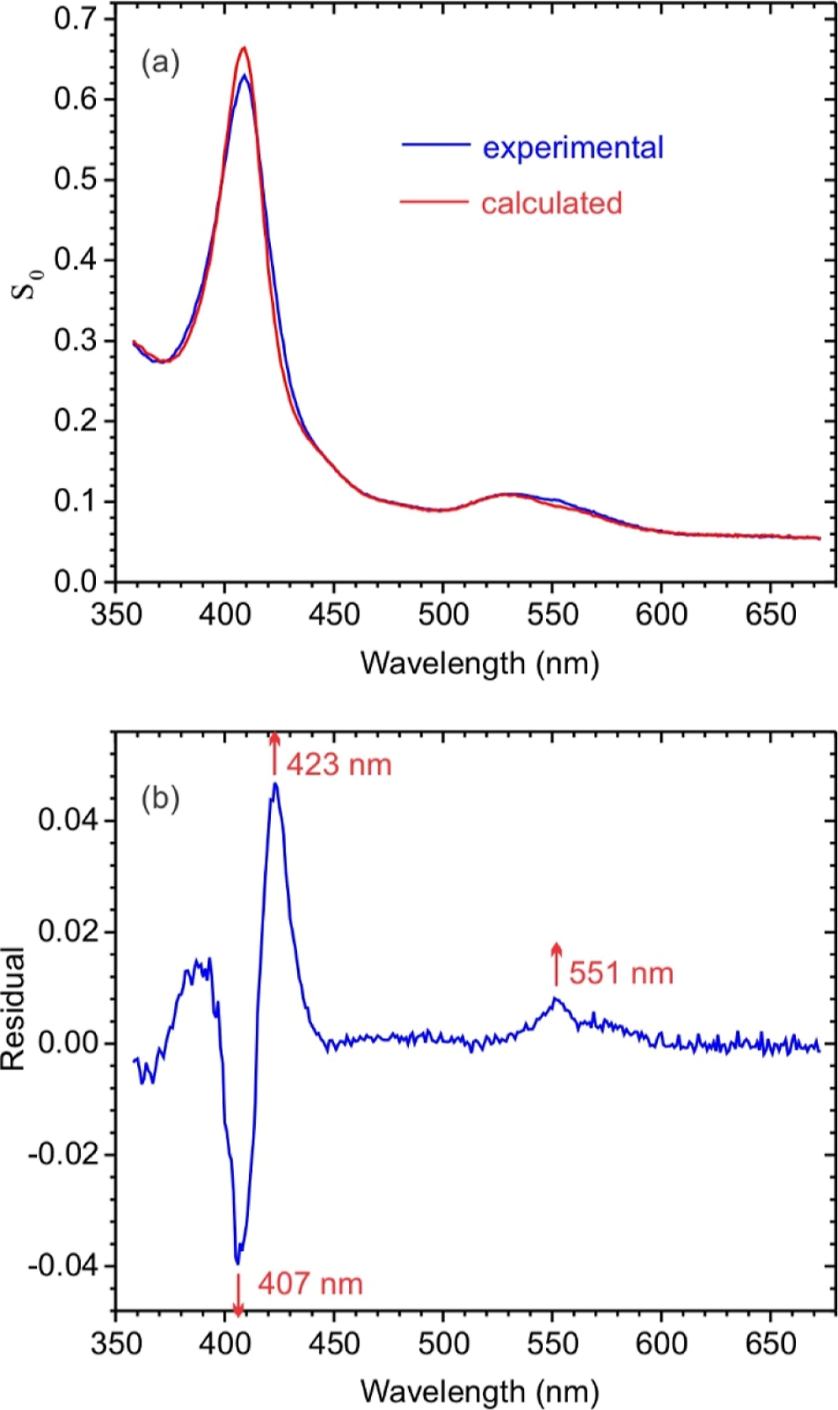
(a) Blue trace: spectral component **S**_**0**_ generated by fitting the SVD-processed Figure 2 data with eq 2a. Red trace: least-squares best fit obtained using the known extinction coefficient spectra of oxidized R103Q and nitrite. The calculated R103Q concentration was 1.15 μM (b) difference spectrum obtained by subtracting the red spectrum in Figure 3a from the blue one.

Though reduction of nitrite-loaded heme 1 in ccNiR_wt_ does not generate a sharp 551 nm peak, it does result in a broad absorbance increase centered around 555 nm, both upon formation of the transient species [Fe_H1_^II^(NO_2_^–^)] (step 1, Scheme 2),^2^ and upon formation of {Fe_H1_NO}^7^ (step 2, Scheme 2).^1^ The residual Figure 3b spectrum exhibits such a broad absorbance increase underlying the sharp 551 nm peak, which suggests that both heme 1 and heme 4 have been reduced within the mixing time and are contributing to the **S**_**0**_ residual.

Figure 4 shows the spectral components (a) **S**_**1**_ and (b) **S**_**2**_ that were obtained by fitting the Figure 2a data with eq 2a. **S**_**1**_ appears with a half-life of about 2 min, while **S**_**2**_ appears with a half-life of about 30 min; neither half-life varies appreciably when the initial Ru^II^ concentration is varied from 200 μM to 1 mM (Figure 5). The **S**_**1**_ and **S**_**2**_ components have broadly similar appearances but exhibit notable small differences. Most importantly, **S**_**1**_ has prominent negative displacements at 524 and 553 nm, together with a noticeable shoulder at 423 nm. These features, which are much less prominent in **S**_**2**_ (the 423 shoulder is absent altogether), are diagnostic for bis-His ligated low-spin heme reoxidation and show that the nonactive site heme (likely heme 4) that reduces within the mixing time then reoxidizes with a half-life of about 2 min. The main features in **S**_**1**_ and **S**_**2**_ are an absorbance increase at 375–380 nm and decrease at ∼410 nm. These features probably indicate the formation of a common species and **S**_**1**_ and **S**_**2**_ are likely spectrally distinct only because the features associated with 6-coordinate low-spin heme reoxidation mix into **S**_**1**_ more than into **S**_**2**_. The amplitude of **S**_**1**_ is only about a quarter that of **S**_**2**_. Importantly, spectral components analogous to **S**_**1**_ and **S**_**2**_ are seen within an hour of exposing nitrite-loaded H257Q and Y206F to mildly reducing conditions (see EPR results below, and also Section S6 of Supporting Information), but not when nitrite-loaded ccNiR_wt_ is exposed to similar conditions.^1,2^ It thus appears that R103, H257, and Y206, together prevent the species with the **S**_**2**_ spectral signature from accumulating in ccNiR_wt_.

**Figure 4:**
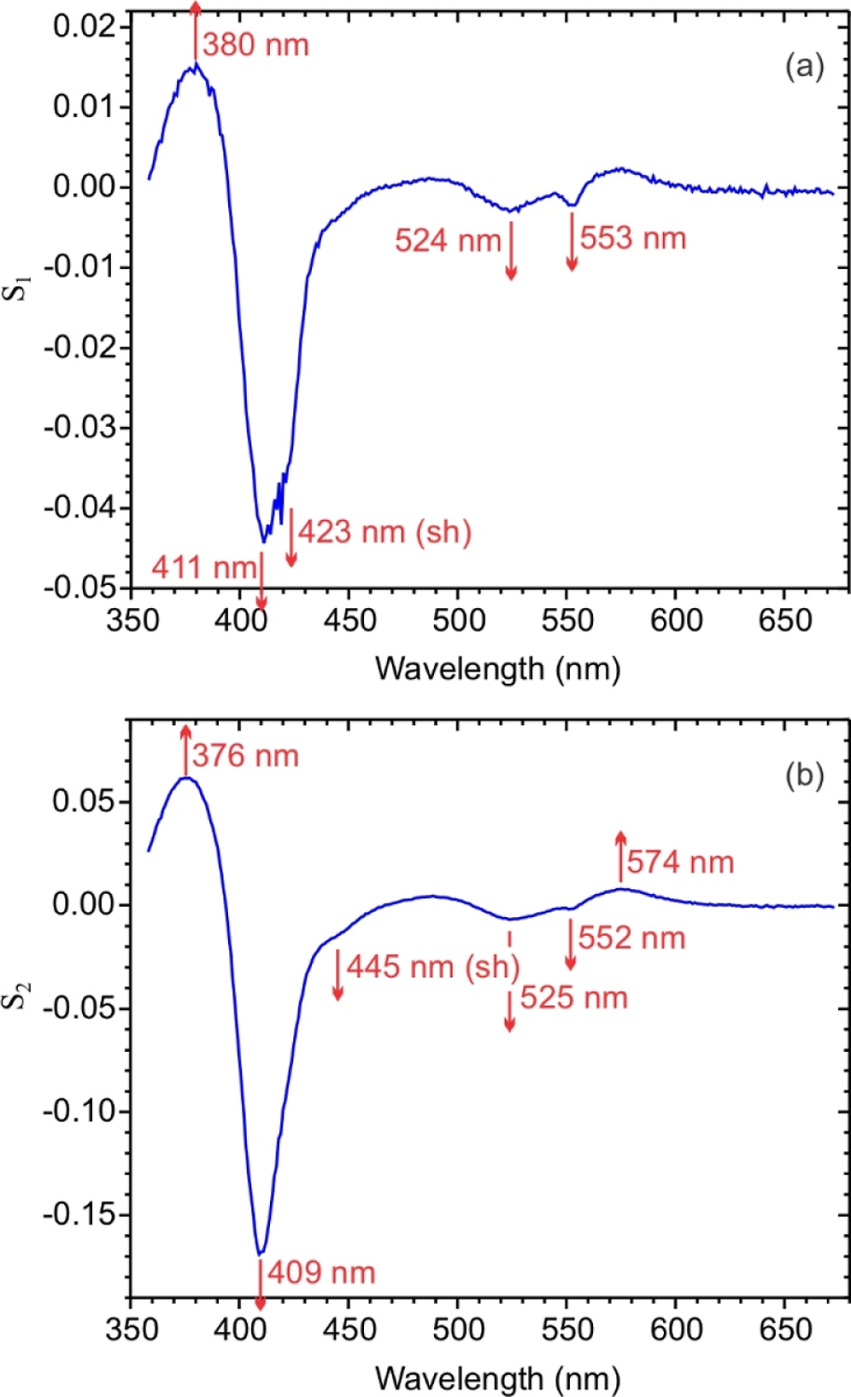
Spectral components (a) **S**_**1**_ and (b) **S**_**2**_ that were obtained by fitting the Figure 2a data to eq 2a.

**Figure 5:**
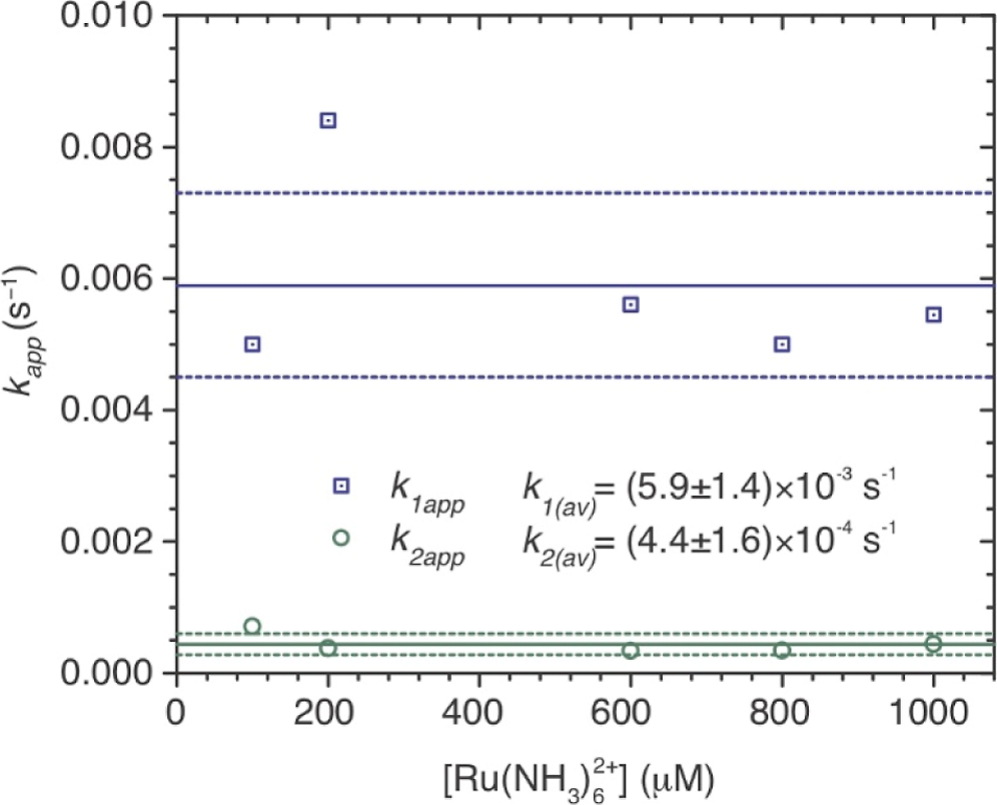
Dependence on initial [Ru^II^] of the *k*_1app_ and *k*_2app_ values obtained from fitting data like those of Figure 2a with eq 2a. In all cases, [NO_2_^–^] = 2 mM and [R103Q] ∼ 0.7 μM.

### EPR Analysis of Nitrite-Loaded R103Q, H257Q, and Y206F after Reduction with Reduced Indigo Tetrasulfonate

We next characterized the species observed in the above UV–vis experiments by electron paramagnetic resonance (EPR) spectroscopy, a technique that is highly sensitive to the coordination environment of the iron where nitrite first coordinates. Hexaammineruthenium­(III) has an EPR spectrum that overlaps with the ccNiR EPR spectra, so for EPR experiments, 2-electron reduced indigo tetrasulfonate (I4S_red_) was used to reduce R103Q. As found with ccNiR_wt_,^2^ the R103Q variant catalyzes reduction of nitrite to NO^•^ by weak reductants (Section S7, Supporting Information). At the high R103Q/I4S_red_ concentration ratios used to prepare the EPR samples, release of NO^•^ from the enzyme resulted in loss of the reduced species with Figure 4 spectral characteristics on a time scale comparable to that of its formation, so the species did not accumulate unless conditions were carefully chosen. Acceptable amounts of the reduced R103Q species with the Figure 4 spectral characteristics could be accumulated by using two equivalents of I4S_red_, but the yield of the species could be roughly doubled by adding to the reaction mixture an excess of 1-(*N,N*-diethylamino)­diazen-1-ium-1,2-diolate (DEANO), which releases NO^•^ in situ at pH 7.^39–41^ The dashed blue trace in Figure 6 shows the spectral change observed 30 min after combining 10 μM R103Q, 2 mM nitrite, and 20 μM reduced I4S, in a 1 mm cuvette, while the red Figure 6 trace shows the difference spectrum from the R103Q species that accumulated after 60 more minutes when 30 equiv of DEANO were added to the initial Figure 6 solution at the 30 min mark. The procedure used for obtaining the Figure 6 spectra is described in detail in Section S8 of Supporting Information but note that both the Figure 6 spectra have comparable shapes to the one seen in spectral components **S**_**1**_ and **S**_**2**_ (Figure 4). The results show that the species generated when I4S_red_ is the electron source is the same one that is produced when Ru^II^ provides the electrons, and that adding NO^•^ to the solution increases the yield of the species when the R103Q/reductant concentration ratio is high. EPR experiments required solutions containing at least 60 μM R103Q and 120 μM I4S_red_. At such high concentrations, the region of the UV–vis spectra between 384 and 422 nm was off scale. However, as shown in Section S8 of Supporting Information, the essential characteristics of the species that gave rise to spectral components **S**_**1**_ and **S**_**2**_ (Figure 4) were still visible in the rest of the UV–vis spectrum.

**Figure 6:**
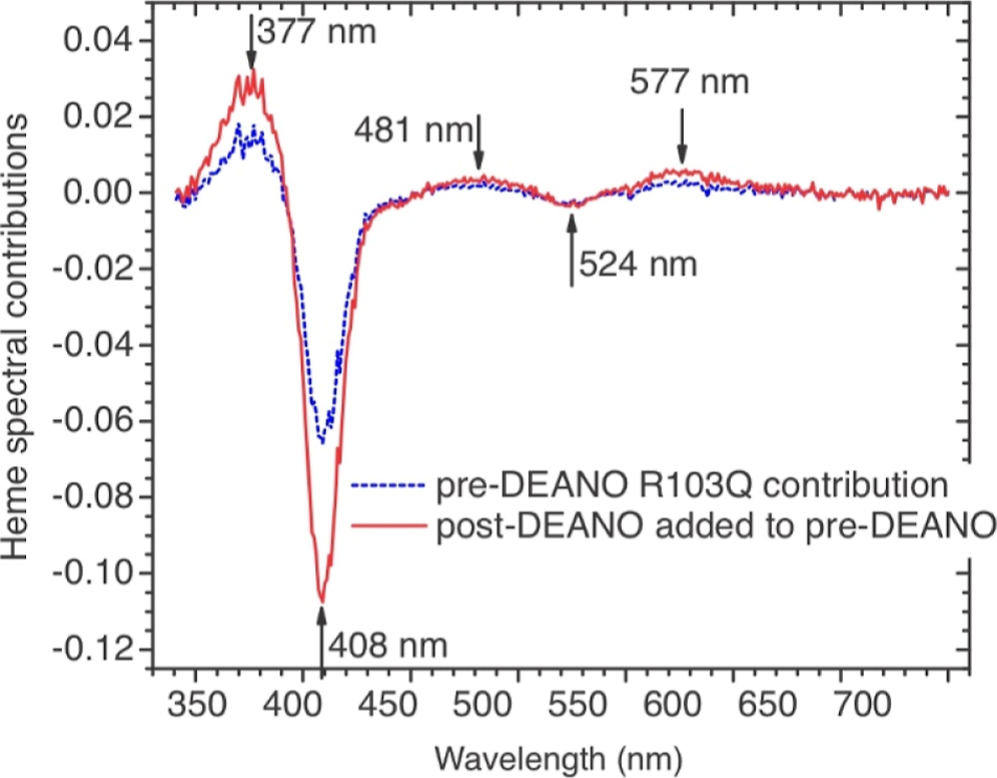
UV–vis spectral changes observed in a 1 mm path length cuvette initially containing 10 μM R103Q, 2 mM nitrite, and 20 μM reduced I4S (pH 7 HEPES buffer), 30 min after mixing the reagents (dashed blue trace), and after adding 30 equiv of the nitric oxide generator DEANO at the 30 min mark and waiting another hour. A detailed description of the procedure used to obtain these difference spectra is provided in Section S8 of Supporting Information.

The blue trace in Figure 7a shows the EPR spectrum between 300 mT and 360 mT obtained at 10 K from a solution initially containing 60 μM R103Q, 2 mM nitrite, and 120 μM I4S_red_, which was allowed to react for 30 min and then frozen at −80 °C. The red trace shows the spectrum obtained in the same field region after adding 30 equiv of DEANO to the same solution at the 30 min mark, allowing the solution to react for a further 60 min, and then freezing it at −80 °C (the full range spectra from 10 mT to 400 mT are provided in Section S9 of Supporting Information). Notably, the EPR spectra were also detectable at 100 K, consistent with a heme nitrosyl species (Figures 7c and S15). Also overlaid in the Figure 7a graph (black trace) is the spectrum of oxidized nitrite-loaded R103Q. The red and blue Figure 7a spectra have virtually identical characteristics, but the spectral intensity after the DEANO addition is substantially greater (approximately two- to 3-fold) than that obtained prior to the addition. Thus, the EPR spectrum behavior mirrors that seen in the UV–vis spectra under the same conditions, demonstrating that it is the EPR spectrum of the species that accumulates within tens of minutes when nitrite-loaded R103Q is exposed to weak reducing agents such as Ru^II^ or I4S_red_. The combined UV–vis and EPR experiments also show that adding a 30-fold excess of NO-generating DEANO to the solution can increase the concentration of the species in question without substantially changing its character.

**Figure 7:**
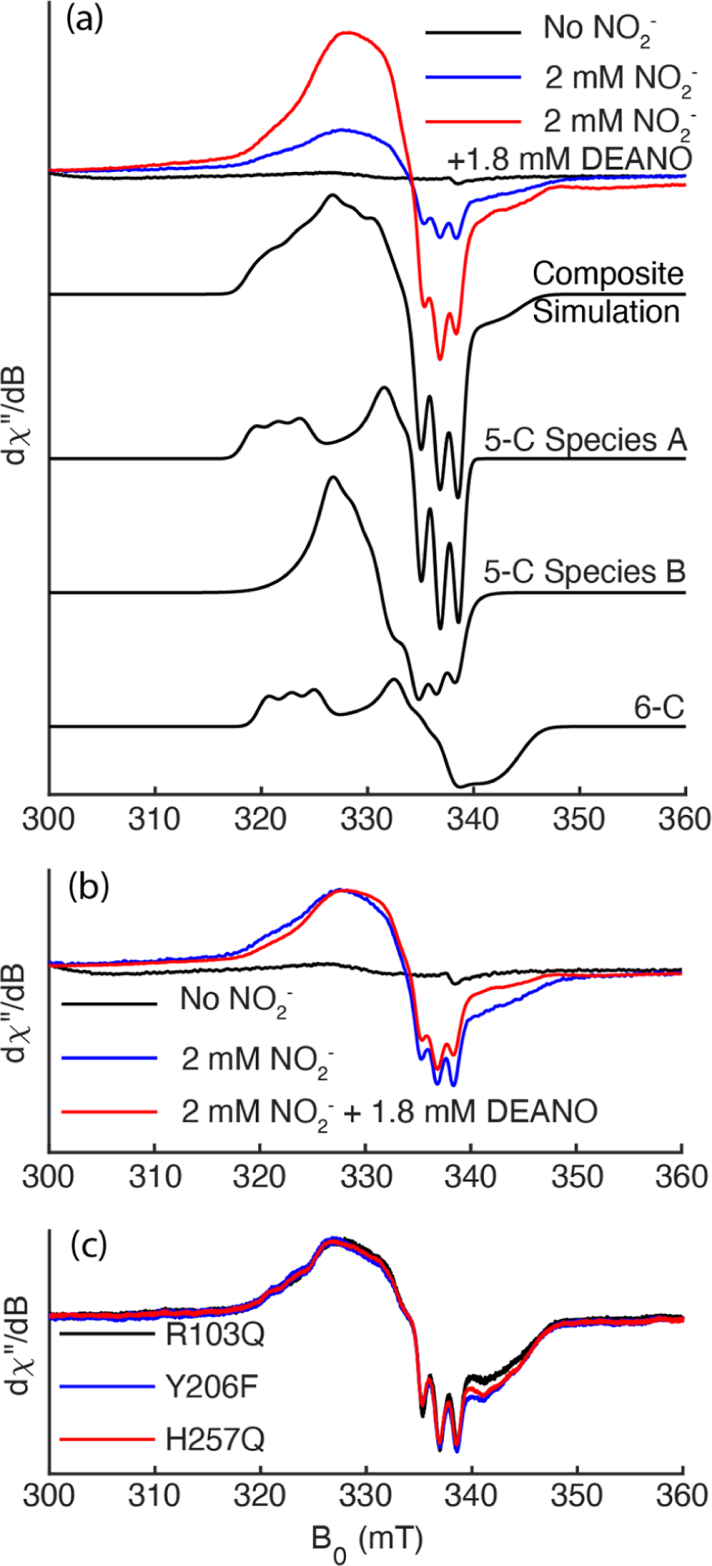
(a) EPR spectrum obtained at 10 K for a solution initially containing 60 μM R103Q, 2 mM nitrite, and 120 μM I4S_red_ before (blue trace) and after (red trace) addition of 30 equiv of the NO^•^ generating species DEANO. The spectrum of the enzyme in the absence of nitrite (black trace) is provided for comparison. Offset black traces: post-DEANO spectrum simulated with two 5-coordinate {FeNO}^7^ species (∼63% total contribution) and one 6-coordinate {FeNO}^7^ species (∼37% contribution). (b) Blue and red traces from (a) scaled to comparable intensity show that the spectrum before DEANO addition may have a somewhat higher contribution from 6-coordinate {FeNO}^7^. (c) Comparison of the EPR spectra obtained at 100 K for nitrite-loaded R103Q, Y206F, and H257Q in the presence of a 2× excess of I4S_red_ and DEANO.

In Figure 7b the EPR spectra obtained before and after DEANO addition have been normalized to the same intensities in the 300 mT region and overlaid to allow their shapes to be easily compared. A narrow spectrum displaying a broad feature with *g*_max_ ≈ 2.07, and a sharp triplet centered at *g* = 2.01, is diagnostic of five-coordinate ferrous-nitrosyl heme complexes at X-band.^51–53^ Indeed, the Figure 7 spectra could be very reasonably simulated by using, without modification, the same ***g-*** and **A**-values (Section S.9, Supporting Information) that were used to simulate at X-band the 5-coordinate ferrous nitrosyl heme EPR spectrum of soluble guanylate cyclase (composite simulation in black, Figure 7a, individual contributions 5-C species A and B).^51^ We note the wing on the spectra present at ∼345 mT. This wing is consistent with a 6-coordinate ferrous nitrosyl heme (Figure 7a, simulation 6-C, also included in the composite simulation). A comparable wing was noted at X-band in the detailed study of the guanylate cyclase heme nitrosyl species, but it was not simulated in that study due to its apparent absence when the same sample was examined at higher frequency (Q-band, 34 GHz).^51,52^ Based on the simulated spectra, we estimate that approximately 37% of the 6-C species is present in the sample with only reductant and NO_2_^–^, and 10% in the sample with DEANO added. The fact that a shift to the blue in the UV–vis heme 1 Soret band accompanies formation of the species (Figures 4 and 6) is also supporting evidence for formation of a 5-coordinate ferrous nitrosyl heme. Typically, *c*-heme reduction is accompanied by a red-shift in the Soret,^1,2^ but when a heme center is reduced to a 5-coordinate ferrous nitrosyl, the Soret typically shifts to the blue.^52,54^ As a final note, the EPR spectrum of the sample obtained in the presence of DEANO was quantified at 100 K, where only the Fe-nitrosyl species is detectable due the fast relaxation of the bis-his coordinated hemes. The results showed approximately 60–70 μM spin concentration in the sample with DEANO added, which is in excellent agreement with the sample concentration, and the expected result if the active site hemes are saturated with NO^•^.^55^

Very importantly, exposing the nitrite-loaded variants H257Q and Y206F to any of the weak reductants TMPD, Ru^II^, or I4S_red_, for tens of minutes, resulted in the same UV–vis and EPR spectral changes as were observed for the R103Q variant. The EPR spectra for R103Q, H257Q and Y206F obtained at 100 K are overlaid in Figure 7c (and Figure S15, Supporting Information) for comparison. By contrast, exposure of nitrite-loaded ccNiR_wt_ to weak reducing agents results in rapid reduction to a form with spectral characteristics very different from those seen for the variants, and no further heme-centered spectral changes beyond the first minute.^1,2^ Thus, it appears that all three active site residues R103, H257, and Y206 must be present to prevent formation, or accumulation, of the 5-coordinate {Fe_H1_NO}^7^ species. As discussed further below, this may be an important function of these residues.

## Discussion

Scheme 3 outlines a minimal mechanism proposed to explain the observed reactivity of nitrite-loaded R103Q, Y206F, or H257Q ccNiR variants with weak reductants. Step 1, the enzyme reduction step, is shown as an equilibrium. This step occurs within the dead time of the R103Q experiments described herein, and the position of equilibrium depends on the reductant used. When fully reduced Ru^II^ is the electron source, the results shown in Figures 2–4 reveal that, in R103Q, both hemes 1 and 4 are at least partially reduced to a detectable extent in this step. Under more weakly reducing conditions such as those provided by a 1:1 mix of Ru^II^ and Ru^III^, reduced R103Q does not accumulate in the short term, suggesting that equilibrium 1 lies far to the left (Section S5, Supporting Information). When ccNiR_wt_ is reduced by even very weak reductants such as TMPD or reduced 1,2-naphthoquinone-4-sulfonic acid, 1-electron reduction of heme 1 is followed within seconds by nitrite dehydration and a second heme 1 reduction, which yields quantitatively a 6-coordinate ferrous nitrosyl, {Fe_H1_NO}^7^ species (Scheme 2).^1–3^ For the variants R103Q, Y206F, or H257Q the corresponding steps would be steps 2 and 3 of Scheme 3, where the ferrous nitrosyl is shown as {Fe_H1_(Lys)­NO}^7^ to indicate that Lys123 is bound at the proximal site (see below). As mentioned above, though, no reduced hemes appear on short time scales when 1:1 Ru^II^/Ru^III^ or weaker reducing conditions are used with R103Q. Similarly, the Y206F and H257Q variants failed to form the 2-electron reduced {Fe_H1_(Lys)­NO}^7^ form when exposed to weak electron donors that readily reduced ccNiR_wt_.^47,56^ These results demonstrate that R103, Y206, and H257 are all needed to facilitate rapid 2-electron reduction of the nitrite-loaded ccNiR active site to the {Fe_H1_(Lys)­NO}^7^ form, at high applied potentials.

**Scheme 3:**
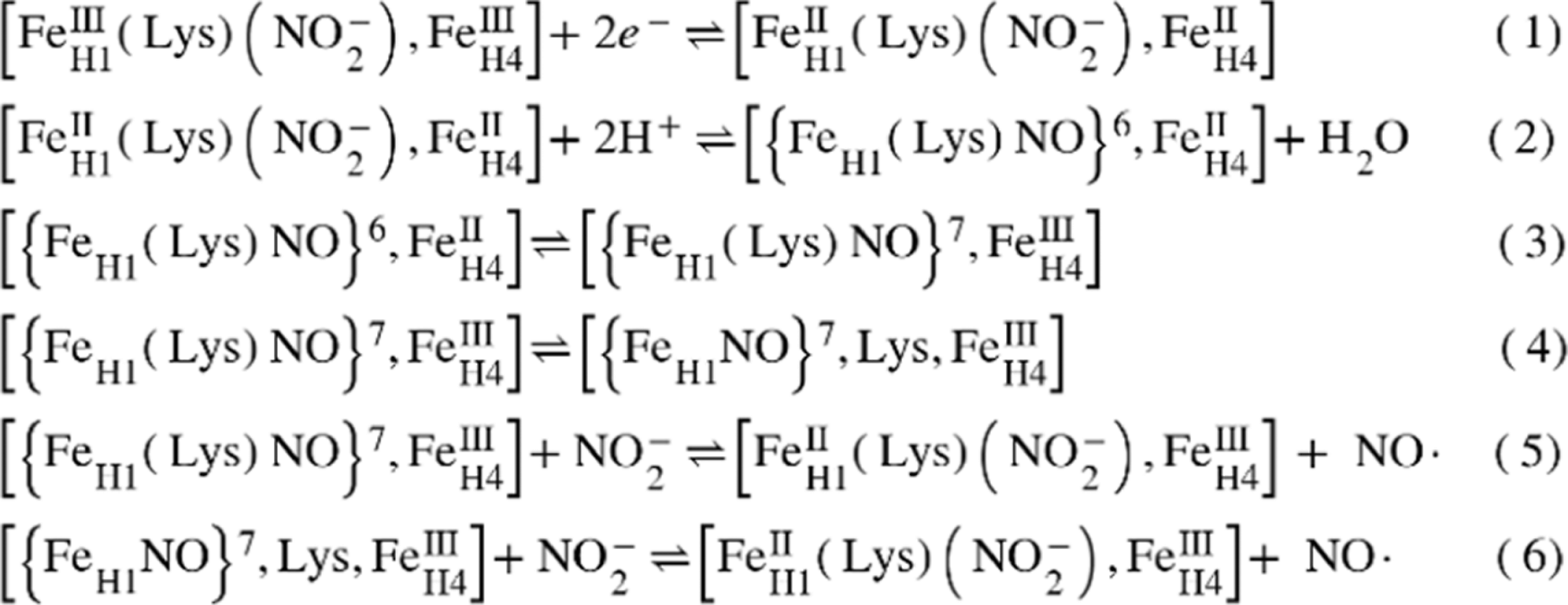
Minimal Mechanism Proposed for the Reactivity of the ccNiR Active Site Variants with Weak Reductants^a^

a {Fe_H1_NO}^7^,Lys represents the active site when Lys 123 has dissociated from the heme. A preliminary stopped-flow experiment showed that when Ru^II^ was used as the electron source, reduction of the bis-His heme pool preceded active site reduction.^4^ When reductant concentration is limited, reactions 5 and 6 will deplete the concentration of 5-coordinate {Fe_H1_NO}^7^ unless excess NO^•^ is added.

When pure Ru^II^ is used as the electron source, the exact nature of the early reduction steps appears to differ from one variant to another. While the Figure 3b residual reveals reduction of both hemes 1 and 4 within the time required to mix nitrite-loaded R103Q with Ru^II^ in conventional UV–vis experiments, a comparable experiment with nitrite-loaded Y206F yields an initial spectral component characteristic of heme 1 reduction to the {Fe_H1_(Lys)­NO}^7^ form but no heme 4 reduction (Figure S5b, Supporting Information). Intriguingly, though, a preliminary stopped-flow experiment with Y206F (not shown)^56^ reveals that heme 4 is reduced within 20 ms of mixing nitrite-loaded Y206F with 1 mM Ru^II^, but that this heme is reoxidized as electrons are transferred to heme 1 (step 3, Scheme 3). A preliminary stopped-flow experiment in which nitrite-loaded R103Q was mixed with 1 mM Ru^II^ also showed that heme 4 reduction preceded active site reduction (data not shown),^4^ but as seen in Figures 3 and 4, in this variant, heme 4 stayed reduced for tens of seconds.

The preliminary stopped-flow experiments with R103Q^4^ and Y206F^56^ revealed further noteworthy differences between variants, and a systematic stopped-flow study of all three variants is currently under way in our laboratories. However, the following definitive conclusions are already apparent regarding the early steps in variant reduction. First, 1 mM pure Ru^II^ can drive an initial equilibrium between Fe_H1_^III^(Lys)­(NO_2_^–^) and {Fe_H1_(Lys)­NO}^7^ (steps 1–3, Scheme 3), at least for R103Q and Y206F. However, this equilibrium lies far to the left compared to the analogous equilibrium for ccNiR_wt_ in the presence of substantially weaker reductants. For example, by comparing the intensity of the Figure S5b difference spectrum, attributed to {Fe_H1_(Lys)­NO}^7^ in Ru^II^-reduced Y206F, with the spectral intensity associated with quantitative conversion of Fe_H1_^III^(Lys)­(NO_2_^–^) to {Fe_H1_(Lys)­NO}^7^ by reduced 1,2-naphthoquinone-4-sulfonic acid,^1^ we estimate that only ∼30% of the Y206F is present as {Fe_H1_(Lys)­NO}^7^ at equilibrium. It is more difficult to estimate the amount of {Fe_H1_(Lys)­NO}^7^ contributing to the Figure 3b spectrum due to R103Q reduction because of the overlapping reduced heme 4 contribution, but it is qualitatively comparable to that seen for Y206F.

In step 4 of Scheme 3, the heme 1 axial ligand Lys123 dissociates from the active site heme 1 to yield the 5-coordinate ferrous nitrosyl. This shifts the equilibrium between Fe_H1_^III^(Lys)­(NO_2_^–^) and the 2-electron reduced nitrosyl-bound active site rightward until the 5-coordinate {Fe_H1_NO}^7^ dominates the EPR spectrum (Figure 7). The equilibrium is complicated by steps 5 and 6 of Scheme 3, which show two ways by which free NO^•^ could be released, leading to catalytic reduction of nitrite to NO^•^ (Section S7, Supporting Information). In step 5, NO^•^ substitution by nitrite takes place at the minority 6-coordinate {Fe_H1_NO}^7^ site, whereas in step 6, the substitution takes place at the 5-coordinate site, after which Lys123 rebinds to the heme. Steps 5 and/or 6 in Scheme 3 can also account for the fact that, when the ratio of reductant to ccNiR variant is low, as it was in the experiments that used I4S_red_ as the electron source, the 5-coordinate {Fe_H1_NO}^7^ species is lost over time unless a source of NO^•^, such as DEANO, is added to push equilibria 5 and 6 to the left. It is well established that a ligand trans to a nitrosyl weakens the Fe–NO bond in heme {FeNO}^7^ species,^57^ so at present we favor step 5 over step 6 in Scheme 3 as the dominant substitution process.

Studies on ccNiR active site variants done prior to this one focused primarily on the mutations’ effects on *k*_cat_ and *K*_m_ values obtained from initial rates in steady-state experiments.^32–34^ In this respect, histidine variants were virtually inactive, the tyrosine variants had about 6% of the wild type’s *k*_cat_ and a 50% smaller *K*_m_, and the arginine variant about 60% of the wild type *k*_cat_ and a 9× greater *K*_m_.^32–34^ The *k*_cat_ and *K*_m_ values that we obtained for *S. oneidensis* R103Q, Y206F, and H257Q using the standard methyl viologen monocation radical assay were in general agreement with previous results (Section S4, Supporting Information). The current study shows that all three active site residues are needed both to stabilize the {Fe_H1_(Lys)­NO}^7^ moiety at high applied potentials and to effect its rapid formation from Fe_H1_^III^(Lys)­(NO_2_^–^) under weakly reducing conditions. These residues also appear to stabilize {Fe_H1_(Lys)­NO}^7^ against loss of the proximal lysine ligand, since the 5-coordinate {Fe_H1_NO}^7^ species is not formed when 2-electron reduced ccNiR_wt_ is allowed to stand for extended periods.^1,2^ The currently accepted mechanism for ccNiR-catalyzed nitrite reduction,^57^ which is based primarily on the computational studies of Bykov et al.,^35–38^ assumes that the proximal lysine stays bound to heme 1 throughout the catalytic cycle. Based on this mechanism, the 5-coordinate {Fe_H1_NO}^7^ species would be off the cycle, and preventing its formation would be an additional important function of R103, Y206, and H257 that has been hitherto unexplored.

The lability of a ligand trans to a ferrous nitrosyl is directly related to the Fe–NO bond strength.^57^ The major contributor to the Fe–NO bond is σ-donation from the singly occupied π* antibonding orbital of the free NO^•^ into the empty d_*z*_^2^ orbital of the Fe, but ligands trans to the nitrosyl compete for the d_*z*_^2^ orbital. Thus, in 6-coordinate nitrosyl complexes, the stronger the Fe–NO bond the weaker the bond of the ligand trans to it, and vice versa. The results presented herein suggest that H257, R103, and Y206 act collectively to make the NO moiety a weaker σ donor, thus allowing for a stronger Fe–N bond from the proximal lysine ligand. When any one of the three active site amino acids is missing, the Fe–NO bond becomes stronger and labilizes the trans Fe–N­(Lys) bond.

The EPR spectrum of nitrite-loaded, 2-electron-reduced ccNiR_wt_ displays several unusual features when compared to other {FeNO}^7^ species reported in the literature.^1^ In our earlier report, we tentatively suggested that these features arise from magnetic interaction of {Fe_H1_NO}^7^ with nearby hemes.^1^ However, as shown in the Figure 7 simulation, the 5-coordinate {Fe_H1_NO}^7^ moiety observed for the variants shows no evidence of magnetic interaction with the other ccNiR hemes. Though this does not rule out such interaction in the wild-type, an alternative explanation that is more consistent with the variant behavior is that the unusual EPR spectrum reported in ref 1 arose from overlapping spectral contributions from atypical rotational conformers of the nitrosyl fragment. Considering the different approaches in generating the {Fe_H1_NO}^7^ species in our previous work with the wild-type enzyme and current results of the variants reported here, we are now further investigating the {Fe_H1_NO}^7^ species in the wild-type with additional EPR experiments and computational analysis.

## Conclusions

To summarize, R103, Y206, and H257 are all needed to stabilize the {Fe_H1_(Lys)­NO}^7^ moiety at high applied potentials, to effect its rapid formation from Fe_H1_^III^(Lys)­(NO_2_^–^) under weakly reducing conditions, and to prevent dissociation of the proximal lysine from heme 1, all of which are likely crucial to optimizing the first steps in ccNiR-catalyzed nitrite reduction. These conclusions are in agreement with earlier protein film voltammetry studies that demonstrated how H257, R103, and Y206 act collectively to enhance the net catalytic reduction of nitrite to ammonium under steady-state conditions.^33^ Thus, while the individual amino acids may have specific roles in certain steps of the catalytic cycle, as suggested by computational studies,^35–38^ their collective role is at least as important. Exactly how R103, Y206, and H257 might act together is an open question, but one plausible hypothesis is that they tune the active site waters. The crystal structures of the various ccNiR homologues reveal several waters in the active site that, together with H257, R103, and Y206 (or their equivalents in other homologues), form an extensive hydrogen bonding network within the active site.^1,24–31^ Perhaps, then, it is the hydrogen bonding network that collectively tailors both the effective formation of {Fe_H1_(Lys)­NO}^7^ from Fe_H1_^III^(Lys)­(NO_2_^–^) and the strength of the Fe–NO bond in the {Fe_H1_NO}^7^ intermediate. Mutation of any one of the three residues H257, R103, or Y206 disrupts the hydrogen bonding network and therefore alters both ccNiR’s catalytic proficiency and its ability to avoid dissociation of the proximal lysine ligand in the {Fe_H1_(Lys)­NO}^7^ catalytic intermediate. Experimental and computational studies are now under way to further explore this hypothesis.

## Supplementary Material



## Notes

Active site ccNiR variant expression vector construction; transformation of the variant plasmids into *S. oneidensis* TSP-C cells; large scale purification of ccNiR active site variants from *S. oneidensis* TSP-C cells; activities of R103Q, H257Q, and Y206F in the standard ccNiR assay; reactivity of nitrite-loaded R103Q ccNiR with 1:1 RuII/RuIII, UV–vis analysis; reactivity of nitrite-loaded Y206F with Ru^II^, UV–vis analysis; R103Q can catalyze nitrite reduction to NO· by TMPD and hexaammineruthenium­(II); reduction of nitrite-loaded R103Q with reduced indigo tetrasulfonate for EPR analysis; parameters used to simulate the reduced variant EPR spectra; full scale EPR spectrum of nitrosylated R103Q; EPR spectra of R103Q, H257Q, and Y206F with and without DEANO at 100 K; expected EPR spectral intensities of the various bis-his hemes compared to the {Fe_H1_NO}^7^ species (PDF)

## References

[1] Ali M., Stein N., Mao Y., Shahid S., Schmidt M., Bennett B., Pacheco A. A. (2019). Trapping
of a putative intermediate
in the cytochrome *c* nitrite reductase (ccNiR)-catalyzed
reduction of nitrite: implications for the ccNiR reaction mechanism. J. Am. Chem. Soc..

[2] Shahid S., Ali M., Legaspi-Humiston D., Wilcoxen J., Pacheco A. A. (2021). A kinetic
investigation of the early steps in cytochrome *c* nitrite
reductase (ccNiR)-catalyzed reduction of nitrite. Biochemistry.

[3] Enemark J. H., Feltham R. D. (1974). Principles of Structure,
Bonding and Reactivity for
Metal Nitrosyl Complexes. Coord. Chem. Rev..

[4] Alam, S. The Effects of an R103Q Mutation on the Chemical and Physical Properties of the Enzyme Cytochrome C Nitrite Reductase (ccNiR); University of Wisconsin-Milwaukee, 2022.

[5] Simon J. (2002). Enzymology
and bioenergetics of respiratory nitrite ammonification. FEMS Microbiol. Rev..

[6] Cole J. A., Brown C. M. (1980). Nitrite Reduction
to Ammonia by Fermentative Bacteria:
A Short Circuit in the Biological Nitrogen Cycle. FEMS Microbiol. Lett..

[7] Poock S., Leach E., Moir J., Cole J., Richardson D. (2002). Respiratory
Detoxification of Nitric Oxide by the Cytochrome *c* Nitrite Reductase of Escherichia coli. J.
Biol. Chem..

[8] Pittman M. S., Elvers K. T., Lee L., Jones M. A., Poole R. K., Park S. F., Kelly D. J. (2007). Growth of Campylobacter jejeuni on
nitrate and nitrite: electron transport to NapA and NrfA via NrfH
and distinct roles for NrfA and the globin Cgb in protection against
nitrosative stress. Mol. Microbiol..

[9] Macuch P. J., Tanner A. C. (2000). Campylobacter species
in health, gingivitis, and periodontitis. J.
Dent. Res..

[10] Klotz M. G., Schmid M. C., Strous M., op den Camp H. J. M., Jetten M. S. M., Hooper A. B. (2008). Evolution of an
octahaem cytochrome
c protein family that is key to aerobic and anaerobic ammonia oxidation
by bacteria. Environ. Microbiol..

[11] Kern M., Volz J., Simon J. (2011). The oxidative
and nitrosative stress
defense network of Wollinella succinogenes: cytochrome *c* nitrite reductase mediates the stress response to nitrite, nitric
oxide, hydroxylamine and hydrogen peroxide. Environ. Microbiol..

[12] Tikhonova T., Tikhonov A., Trofimov A., Polyakov K., Boyko K., Cherkashin E., Rakitina T., Sorokin D., Popov V. (2012). Comparative
structural and functional analysis of two octaheme nitrite reductases
from closely related Thioalkalovibrio species. FEBS J..

[13] Canfield D. E., Glazer A. N., Falkowski P. G. (2010). The Evolution
and Future of Earth’s
Nitrogen Cycle. Science.

[14] Bernhard A. E. (2010). The nitrogen
cycle: processes, players, and the human impact. Nat. Educ. Knowl..

[15] Raymond J., Siefert J. L., Staples C. R., Blankenship R. E. (2004). The natural
history of nitrogen fixation. Mol. Biol. Evol..

[16] Da Silva, J. F. ; Williams, R. J. P. The Biological Chemistry of the Elements: The Inorganic Chemistry of Life; Oxford University Press, 2001.

[17] Bothe, H. ; Ferguson, S. J. ; Newton, W. E. Biology of the Nitrogen Cycle; Elsevier: Amsterdam, 2007.

[18] Moreno-Vivián C., Ferguson S. J. (1998). Definition and distinction between assimilatory, dissimilatory,
and respiratory pathways. Mol. Microbiol..

[19] Galloway J. N., Leach A. M., Bleeker A., Erisman J. W. (2013). A chronology of
human understanding of the nitrogen cycle. Philos.
Trans. R. Soc., B.

[20] Galloway J. N., Townsend A. R., Erisman J. W., Bekunda M., Cai Z., Freney J. R., Martinelli L. A., Seitzinger S. P., Sutton M. A. (2008). Transformation of the Nitrogen Cycle:
Recent Trends,
Questions and Potential Solutions. Science.

[21] Duce R. A., LaRoche J., Altieri K., Arrigo K. R., Baker A. R., Capone D. G., Cornell S., Dentener F., Galloway J., Ganeshram R. S., Geider R. J., Jickells T., Kuypers M. M., Langlois R., Liss P. S., Liu S. M., Middelburg J. J., Moore C. M., Nickovic S., Oschlies A., Pedersen T., Prospero J., Schlitzer R., Seitzinger S., Sorensen L. L., Uematsu M., Ulloa O., Voss M., Ward B., Zamora L. (2008). Impacts of Atmospheric
Anthropogenic
Nitrogen on the Open Ocean. Science.

[22] Ravishankara A. R., Daniel J. S., Portmann R. W. (2009). Nitrous oxide (N_2_O): the
dominant ozone-depleting substance emitted in the 21st century. Science.

[23] Wuebbles D. J. (2009). Nitrous
oxide: no laughing matter. Science.

[24] Einsle O., Messerschmidt A., Stach P., Bourenkov G. P., Bartunik H. D., Huber R., Kroneck P. M. H. (1999). Structure of
cytochrome c nitrite reductase. Nature.

[25] Einsle O., Stach P., Messerschmidt A., Simon J., Kroger A., Huber R., Kroneck P. M. H. (2000). Cytochrome
c nitrite reductase from
Wolinella succinogenes—Structure at 1.6 angstrom resolution,
inhibitor binding, and heme-packing motifs. J. Biol. Chem..

[26] Bamford V. A., Angove H. C., Seward H. E., Thomson A. J., Cole J. A., Butt J. N., Hemmings A. M., Richardson D. J. (2002). Structure
and spectroscopy of the periplasmic cytochrome c nitrite reductase
from Escherichia coli. Biochemistry.

[27] Cunha C. A., Macieira S., Dias J. M., Almeida G., Goncalves L. L., Costa C., Lampreia J., Huber R., Moura J. J. G., Moura I., Romao M. J. (2003). Cytochrome
c nitrite reductase from
Desulfovibrio desulfuricans ATCC 27774—The relevance of the
two calcium sites in the structure of the catalytic subunit (NrfA). J. Biol. Chem..

[28] Rodrigues M., Oliveira T., Pereira I., Archer M. (2006). X-ray structure of
the membrane-bound cytochrome *c* quinol dehydrogenase
NrfH reveals novel haem coordination. EMBO J..

[29] Youngblut M., Judd E. T., Srajer V., Sayyed B., Goelzer T., Elliott S. J., Schmidt M., Pacheco A. A. (2012). Laue crystal
structure
of Shewanella oneidensis cytochrome *c* nitrite reductase
from a high-yield expression system. J. Biol.
Inorg. Chem..

[30] Campecino J., Lagishetty S., Wawrzak Z., Sosa Alfaro V., Lehnert N., Reguera G., Hu J., Hegg E. (2020). Cytochrome
c nitrite reductase from the bacterium Geobacter lovleyi represents
a new NrfA subclass. J. Biol. Chem..

[31] Denkhaus L., Siffert F., Einsle O. (2023). An unusual active site
architecture
in cytochrome *c* nitrite reductase NrfA-1 from Geobacter
metallireducens. FEMS Microbiol. Lett..

[32] Lukat P., Rudolf M., Stach P., Messerschmidt A., Kroneck P., Simon J., Einsle O. (2008). Binding and Reduction
of Sulfite by Cytochrome *c* Nitrite Reductase. Biochemistry.

[33] Judd E. T., Stein N., Pacheco A. A., Elliott S. J. (2014). Hydrogen bonding
networks tune proton-coupled redox steps during the enzymatic six-electron
conversion of nitrite to ammonia. Biochemistry.

[34] Lockwood C. W. J., Burlat B., Cheesman M. R., Kern M., Simon J., Clarke T., Richardson D. J., Butt J. N. (2015). Resolution of key
roles for the distal pocket histidine in cytochrome *c* nitrite reductases. J. Am. Chem. Soc..

[35] Bykov D., Neese F. (2011). Substrate binding and activation in the active site of cytochrome *c* nitrite reductase: a density functional study. J. Biol. Inorg. Chem..

[36] Bykov D., Neese F. (2012). Reductive activation
of the heme iron-nitrosyl intermediate in the
reaction mechanism of cytochrome c nitrite reductase: a theoretical
study. J. Biol. Inorg. Chem..

[37] Bykov D., Plog M., Neese F. (2014). Heme-bound
nitroxyl, hydroxylamine,
and ammonia ligands as intermediates in the reaction cycle of cytochrome
c nitrite reductase: a theoretical study. J.
Biol. Inorg. Chem..

[38] Bykov D., Neese F. (2015). Six-electron reduction
of nitrite to ammonia by cytochrome *c* nitrite reductase:
insights from density functional theory
studies. Inorg. Chem..

[39] Purwar N., McGarry J. M., Kostera J., Pacheco A. A., Schmidt M. (2011). Interaction
of nitric oxide with catalase: structural and kinetic analysis. Biochemistry.

[40] Drago R. S., Paulik F. E. (1960). The Reaction of
Nitrogen (II) Oxide with Diethylamine. J. Am.
Chem. Soc..

[41] Maragos C. M., Morley D., Wink D. A., Dunams T. M., Saavedra J. E., Hoffman A., Bove A. A., Isaac L., Hrabie J. A., Keefer L. K. (1991). Complexes of NO
with Nucleophiles as Agents for the
Controlled Biological Release of Nitric-Oxide—Vasorelaxant
Effects. J. Med. Chem..

[42] Watanabe T., Honda K. (1982). Measurement of the extinction coefficient
of the methyl viologen
cation radical and the efficiency of its formation by semiconductor
photocatalysis. J. Phys. Chem..

[43] Koebke K. J., Pauly D. J., Lerner L., Liu X., Pacheco A. A. (2013). Does the
oxidation of nitric oxide by oxymyoglobin share an intermediate with
the metmyoglobin-catalyzed isomerization of peroxynitrite?. Inorg. Chem..

[44] Koebke K. J., Waletzko M. T., Pacheco A. A. (2016). Direct
monitoring of the reaction
between photochemically generated nitric oxide and Mycobacterium tuberculosis
tuncated hemoglobin N wild type and variant forms: an assessment of
computational mechanistic predictions. Biochemistry.

[45] Aasa R., Vanngard T. (1975). EPR signal intensity
and powder shapes: a reexamination. J. Magn.
Reson..

[46] Stoll S. A. S., Schweiger A. (2006). EasySpin,
a comprehensive software package for spectral
simulation and analysis in EPR. J. Magn. Reson..

[47] Shahid, S. A Mechanistic Investigation of Cytochrome C Nitrite Reductase Catalyzed Reduction of Nitrite to Ammonia: The Search for Catalytic Intermediates; University of Wisconsin-Milwaukee: Milwaukee, WI, 2020.

[48] Arciero D. M., Collins M. J., Haladjian J., Bianco P., Hooper A. B. (1991). Resolution
of the 4 Hemes of Cytochrome-C554 from Nitrosomonas-Europaea by Redox
Potentiometry and Optical Spectroscopy. Biochemistry.

[49] Marritt S. J., Kemp G. L., Xiaoe L., Durrant J. R., Cheesman M. R., Butt J. N. (2008). Spectroelectrochemical
characterization of a pentaheme
cytochrome in solution and as electrocatalytically active films on
nanocrystalline metal-oxide electrodes. J. Am.
Chem. Soc..

[50] Stein N., Love D., Judd E. T., Elliott S. J., Bennett B., Pacheco A. A. (2015). Correlations between
the electronic properties of *Shewanella oneidensis* cytochrome *c* nitrite
reductase (ccNiR) and its structure: effects of heme oxidation state
and active site ligation. Biochemistry.

[51] Gunn A., Derbyshire E. R., Marletta M. A., Britt R. D. (2012). Conformationally
distinct five-coordinate heme-NO complexes of soluble guanylate cyclase
elucidated by multifrequency electron paramagnetic resonance (EPR). Biochemistry.

[52] Martin E., Berka V., Sharina I., Tsai A.-L. (2012). Mechanism of binding
of NO to soluble guanylyl cyclase: implication for the second NO binding
to the heme proximal site. Biochemistry.

[53] Stone J. R., Sands R. H., Dunham W. R., Marletta M. A. (1995). Electron paramagnetic
resonance spectral evidence for the formation of a pentacoordinate
nitrosyl-heme complex on soluble guanylate cyclase. Biochem. Biophys. Res. Commun..

[54] Stone J. R., Marletta M. A. (1994). Soluble guanylate cyclase from bovine lung: activation
with nitric oxide and carbon monoxide and spectral characterization
of the ferrous and ferric states. Biochemistry.

[55] Motion C. L., Lovett J. E., Bell S., Cassidy S. L., Cruickshank P. A. S., Bolton D. R., Hunter R. I., El Mkami H., Van Doorslaer S., Smith G. M. (2016). DEER sensitivity between iron centers
and nitroxides
in heme containing proteins improves dramatically using broadband
high-field EPR. J. Phys. Chem. Lett..

[56] Reinhardt, S. J. The Effects of Active Site Mutations on the Chemical and Physical Properties of the Enzyme Cytochrome C Nitrite Reductase; University of WIsconsin-Milwaukee, 2025.

[57] Lehnert N., Kim E., Dong H. T., Harland J. B., Hunt A. P., Manickas E. C., Oakley K. M., Pham J., Reed G. C., Alfaro V. S. (2021). The biologically
relevant coordination chemistry of iron and nitric oxide: electronic
structure and reactivity. Chem. Rev..

