# Supplementary material for: Roles of Three Conserved Active Site Residues of Cytochrome c Nitrite Reductases During the Early Steps of Nitrite Reduction
Source: J Am Chem Soc. 2025 Jul 23;147(31):27355–66. doi: 10.1021/jacs.5c03297 (PMC12498419; doi:10.1021/jacs.5c03297)
Supplement: Supplementary file 1 [file ja5c03297_si_001.pdf]

Supporting Information for:

Roles of Three Conserved Active Site Residues of Cytochrome *c* Nitrite Reductases During the Early Steps of Nitrite Reduction

Shahama Alam,[‡,◊] Bradley Dimock,[‡,◊] Brian Bennett,[†] Steven J. Reinhardt,[‡] Shahid Shahid,[‡] A. Andrew Pacheco,[*,‡] and Jarett Wilcoxen[*,‡]

* Corresponding authors

[‡] Department of Chemistry and Biochemistry, University of Wisconsin-Milwaukee, Milwaukee, Wisconsin 53211, United States

[†] Department of Physics, Marquette University, 1420 W. Clybourn St., Milwaukee, Wisconsin, 53233, United States

[◊] These authors contributed equally

S1. Active site ccNiR variant expression vector construction.

R103Q, H257Q, and Y206F variants of *S. oneidensis* ccNiR were prepared starting from the wild type expression system originally reported by Youngblut et al.[2] This expression system consists of the small tetraheme *c* leader sequence fused to the N-terminal of the wild type ccNiR gene, inserted into a pHSG299 plasmid, and transformed into TSP-C *Shewanella oneidensis* (an MR-1-like strain of *S. oneidensis* resistant to rifampicin).[2] To make the desired variants separable from wild type ccNiR expressed genomically by *S. oneidensis*, the original expression system was further modified by adding to the C-terminal of the gene a TEV cleavage site, a glycine linker, a 10× histidine tag, and a two-amino-acid cap (ThrGly). Initially, the His-tagged wild type gene was made by GenScript but provided in a pJET1.2 plasmid that is not suitable for protein expression. The gene was amplified by PCR using suitable primers and cloned into pHSG299 (a PUC-type vector with a kanamycin resistance gene). NEB5-α *E. coli* were transformed per manufacturer's instructions, plated on LB agar plates containing 50 mg/mL of kanamycin, and grown for 24 hours at 37 °C. Colonies were picked, placed into 5 mL of LB with 50 mg/mL of kanamycin and grown for 12-14 hours. Colony PCR was performed using M13 forward and reverse primers and run on an agarose gel to verify the presence of an insert of the correct size (~1500 bp). Aliquots of cultures with the correctly sized insert were harvested using a plasmid miniprep kit (Qiagen), after which sequencing at the University of Chicago Comprehensive Cancer Center confirmed that the plasmids contained the 10× his-tagged wild type ccNiR gene. Aliquots of the plasmid-containing *E. coli* culture were preserved in 10% glycerol and stored at −80 °C for cell stock.

The desired active site variants were prepared from the his-tagged gene using the following primers. For R103Q: GTAATACCCTA<u>CAA</u>ACCGGCGCACC (forward), GAACATCGGTCACAGCATACATATGACC (reverse); for H257Q: CTTAAAGCACAG<u>CAA</u>CCTGAATACGAG (forward), CATTGGGGTCTTACTAAGGGCATGG (reverse); for Y206F: GCAGTGCCATGTTGAA<u>TTT</u>TACTTTG (forward), GCACACACCATACTTTCTTTATCTTGC (reverse). In each case, the substituted codon is underlined on the forward primer. All primers contained a 5´-phosphate to allow blunt-end ligation. Gel electrophoresis of the PCR products showed 4.2 kb bands, which are the correct size for the ccNiR-containing plasmids. These bands were cut out and cleaned using a gel cleaning kit (Qiagen) to obtain linear plasmid using the manufacturer's protocol. The instant sticky-end ligase master mix (5 µL, New England Biolabs) was used to ligate the linear plasmids (5 µL) and make them circular. The circular plasmids were then transformed into chemically competent NEB5-α cells (New England Biolabs) following the manufacturer's protocol. To select for plasmid-containing cells, the bacteria were cultured overnight on LB and kanamycin (50 µg/mL) containing petri dishes at 37 °C. A few single colonies were picked and grown separately in mini culture (5 mL LB with 50 µg/mL kanamycin) to isolate the plasmid. This plasmid was used as a template for PCR using M13 forward and reverse primers. The PCR products of the right size were again identified by gel electrophoresis, cleaned using a PCR clean-up kit (Qiagen), and sent to the University of Chicago Comprehensive Cancer Center for sequencing. The sequencing confirmed that the isolated plasmids contained the desired ccNiR variants.

S2. Transformation of the variant plasmids into *S. oneidensis* TSP-C cells.

Plasmids containing the variant ccNiR genes were transferred to the TSP-C strain of *S. oneidensis* by electroporation. TSP-C cells were grown overnight (~16 hours) at 30 °C with 30 µg/mL rifampicin. Approximately 2 mL of overnight culture were spun at 4300 × g to obtain a cell pellet, washed with 500 µL 1 M sorbitol, decanted, and then resuspended in 80 µL 1M sorbitol. A 20 µL aliquot of the desired

variant ccNiR plasmid was added to the resuspended cells, which were then electroporated at 1500 V, mixed with 800 µL of SOC growth medium, and incubated at 30 °C for 1-2 hours at 200 rpm in an incubator shaker. The newly transformed cells were incubated overnight at 30 °C on a plate containing LB, kanamycin (50 µg/mL), and rifampicin (30 µg/mL). Only positively transformed bacteria grew on this plate, and they appeared as pink colonies. To further confirm the result, colony PCR was also performed, and the PCR products were analyzed by gel electrophoresis. New 1.5 kb bands on the gels confirmed the presence of the bacterial plasmids that contained the variants. After this confirmation, the bacterial colonies from the petri dish were further cultured and then stored at –80 °C in 10% glycerol for future experiments.

S3. Large scale purification of ccNiR active site variants from *S. oneidensis* TSP-C cells.

A petri dish containing LB agarose, kanamycin (Kan, 50 µg/mL), and rifampicin (Rif, 30 µg/mL), was inoculated with *S. oneidensis* TSP-C cells containing the desired ccNiR gene and incubated overnight at 30 °C. A single bacterial colony from the petri dish was suspended into 5 mL LB (20 mg/mL), Kan (50 µg/mL), Rif (30 µg/mL) solution and was incubated for 10-11 hours in an incubator shaker at 200 rpm and 30 °C. After that period, 1 mL of bacterial culture was transferred into 1 L of the same medium, which was then incubated for 16 hours under the same conditions as in the first step. In a final step, 1 L of bacterial cell culture was transferred into a 50 L carboy that already contained 45 L LB, Kan (50 µg/mL), Rif (30 µg/mL) thermostated at 30 °C in a constant temperature water bath. The culture was incubated for 16 - 18 hours at 30 °C while being continually sparged with compressed air, which served to agitate the culture suspension and to keep it aerated until late in the growth process when high bacterial density consumed oxygen faster than it could be replenished. The cells were harvested from the 45 L cell culture by centrifuging 1 L aliquots for 10 minutes at 3,800 × g using the Bioflex HC rotor. The pooled cell pellets were resuspended using 20 mM tris buffer, pH = 8.1 (final volume ~500 mL), and either PMSF (0.1 - 1 mM) or AEBSF (0.1 – 1 mM) was immediately added as a protease inhibitor. The resuspended pooled cell pellet was frozen and stored at −80 °C in a stainless-steel beaker until needed.

For purification, a cell suspension containing the desired ccNiR variant was thawed, then lysed by sonication in a water/ice-cooled stainless-steel beaker, using cycles of 30 s ultrasonic bursts at 70% amplitude followed by 45 s pauses, repeated for 10 minutes. The suspension was then centrifuged at 30,000 × g for 60 minutes using the F14-6x250y rotor to remove cell debris, and the clarified supernatant was separated. Imidazole was dissolved into the supernatant to 40 mM, which was then filtered using a 0.2 µM syringe filter and loaded onto an affinity column (GE Healthcare HisTrap FF, 20 mL). This column was pre-equilibrated with a buffer (Buffer A) that contained 40 mM imidazole, 20 mM tris base, and 500 mM NaCl, adjusted to pH = 8.1. After the clarified cell extract had been loaded, the column was washed with 5-10 column volumes of Buffer A until the UV reading at 280 nm of the column flow-through was once again constant. The desired his-tagged variant was eluted by mixing Buffer A with 80% of a pH 7.0 buffer containing 500 mM Imidazole, 20 mM HEPES, and 500 mM NaCl (Buffer B). All fractions for which the A280 deviated significantly from the baseline were pooled and immediately transferred to Snake-Skin dialysis tubes (9 mL /1 cm, ThermoFisher) for roughly 4 hours of dialysis at 4 °C in a low salt buffer reservoir (20 mM HEPES, 500 mM NaCl, pH = 7.0; Buffer C). The His-tag was removed by incubating the protein with tobacco etch virus (TEV) protease. TEV protease was added to the dialysis tube containing the desired variant according to the rule of thumb protocol of adding 1.0 O.D. TEV protease to 10 O.D mutant ccNiR. The dialysis tube was then transferred into another buffer

reservoir (50 mM Tris, 100 mM NaCl, 1 mM DTT, 250 µM EDTA, pH = 8.0; Buffer D) which is an optimum buffer for TEV protease activity. The digestion of His-tag by TEV protease continued overnight at 4 °C. The digested protein solution was centrifuged at 39,000 × g for 10 min using the F20-12x50 rotor and the supernatant was collected. A HisTrap FF column (5 mL, GE Healthcare) was equilibrated with a buffer containing 25 mM tris, 300 mM NaCl, pH = 8.0 (Buffer E), after which the clarified protein solution was loaded onto it. This column captured undigested variant ccNiR and the TEV protease, which is also His-tagged, but allowed the variant ccNiR from which the His-tag had been successfully cleaved to pass through. The buffer from the flow-through was exchanged for a buffer containing 50 mM HEPES, 150 mM NaCl, pH = 7.0 (Buffer F), and then concentrated using centrifugal concentrators (Amicon) spun at 4,000 g for 10 min intervals. Size exclusion chromatography (SEC, Sephacryl-S200, GE Healthcare) was used as the final step of purification. The SEC column (320 mL) was equilibrated with Buffer F and then loaded with a concentrated variant ccNiR sample (not more than 2.5 mL). Two distinct UV280 peaks were observed in the chromatograph. The second of these typically displayed UV-vis ratios A409/A280 > 3.8 and was confirmed to be the pure variant ccNiR by SDS-PAGE. The pure protein's buffer was exchanged for 50 mM HEPES at pH = 7.0, after which the solution was concentrated using centrifugal concentrators and stored at –80 °C.

S4. Activities of R103Q, H257Q, and Y206F in the standard ccNiR assay

The abilities of the *S. oneidensis* ccNiR variants R103Q, H257Q, and Y206F to catalyze reduction of nitrite and hydroxylamine with the methyl viologen monocation radical (MV_{red}) were measured and compared to those of the wild type enzyme. Scheme S1 shows the stoichiometry of the nitrite reduction process and defines the parameters obtained in the assay, While Scheme S2 does the same for the hydroxylamine reduction process. Tables S1 and S2 summarize the parameter values obtained in the two assays for the R103Q, H257Q, and Y206F variants and compare them to those of the wild type enzyme. The results mirror those obtained earlier using protein film voltammetry.[3]

$$6MV_{red} + NO_2^- + 8H^+ \xrightarrow{ccNiR} 6MV_{ox} + NH_4^+ + 2H_2O$$

$$\frac{V_{01}}{[ccNiR]} = \frac{k_{kat1}[NO_2^-]}{K_{m1} + [NO_2^-]}$$

Scheme S1. Standard ccNiR assay for nitrite reduction. V_{01} represents the initial rate of MV_{red} oxidation (in M/s).

Table S1. Parameters k_{cat1} and K_{m1} (Scheme S1) obtained for nitrite reduction by MV_{red} for wild type ccNiR and its variants.

	k_{cat1} (s^{-1})	K_{m1} (M)
Wild type	4510 ± 90	$(2.4 \pm 0.6) \times 10^{-5}$
R103Q	780 ± 50	$(4.7 \pm 0.9) \times 10^{-4}$
H257Q	11.3± 0.6	$(1.5 \pm 0.3) \times 10^{-4}$
Y206F	93 ± 5	$(2.2 \pm 0.5) \times 10^{-5}$

$$2MV_{red} + NH_2OH + 3H^+ \xrightarrow{ccNiR} 2MV_{ox} + NH_4^+ + H_2O$$

$$\frac{V_{02}}{[ccNiR]} = \frac{k_{kat2}[NH_2OH]}{K_{m2} + [NH_2OH]}$$

Scheme S2. Standard ccNiR assay for hydroxylamine reduction. V_{02} represents the initial rate of MV_{red} oxidation (in M/s).

Table S2. Parameters k_{cat2} and K_{m2} (Scheme S2) obtained for hydroxylamine reduction by MV_{red} for wild type ccNiR and its variants.

	k_{cat1} (s^{-1})	K_{m1} (M)
Wild type	3110 ± 300	$(2.1 \pm 0.7) \times 10^{-2}$
R103Q	600 ± 30	$(2.7 \pm 0.6) \times 10^{-2}$
H257Q	2050± 140	$(2.1 \pm 0.5) \times 10^{-2}$
Y206F	285 ± 12	$(1.6 \pm 0.7) \times 10^{-3}$

S5. Reactivity of nitrite-loaded R103Q ccNiR with 1:1 Ru^{II}:Ru^{III}, UV/Vis analysis

Figure S1a shows the UV/Vis spectral changes observed after mixing 1.6 μM R103QccNiR with 2 mM nitrite and a 1:1 mixture of Ru^{II}:Ru^{III} (600 μM total ruthenium) in a pH 7 HEPES buffer. The Ru^{II}:Ru^{III} mixture was generated electrochemically by applying a potential of 50 mV vs SHE to a stock solution of Ru^{III} (50 mV is the midpoint potential for Ru^{III} reduction). SVD analysis showed that only two spectral components were needed to faithfully reconstruct a noise-reduced absorbance matrix. The SVD-treated data were then fit to Eq. S1 using a global fitting routine (red traces, Fig. S1a).[4, 5] In Eq.S1, $A_{\lambda,t}$ is the absorbance obtained at wavelength λ and

$$A_{\lambda,t} = S_{3(\lambda)} + S_{4(\lambda)}\left[1 - \exp(-k_{app3}t)\right] \quad \text{Eq. S1}$$

time t, $\mathbf{S_3}$ is the spectral component present at t = 0, and $\mathbf{S_4}$ is a spectral component that grows in exponentially at a rate governed by k_{app3}. As explained for $\mathbf{S_0}$ in the main text, $\mathbf{S_3}$ should decay exponentially as $\mathbf{S_4}$ grows in, at a rate governed by k_{ap3}, but when $\mathbf{S_3}$ is treated as a constant, $\mathbf{S_4}$ will appear as a difference spectrum. Figure S1b shows an absorbance vs. time slice from the Fig. S1a data set, obtained at 409 nm, the Soret maximum for R103Q ccNiR. The blue trace is experimentally obtained, the red one is the least-squares best fit to Eq. S1.

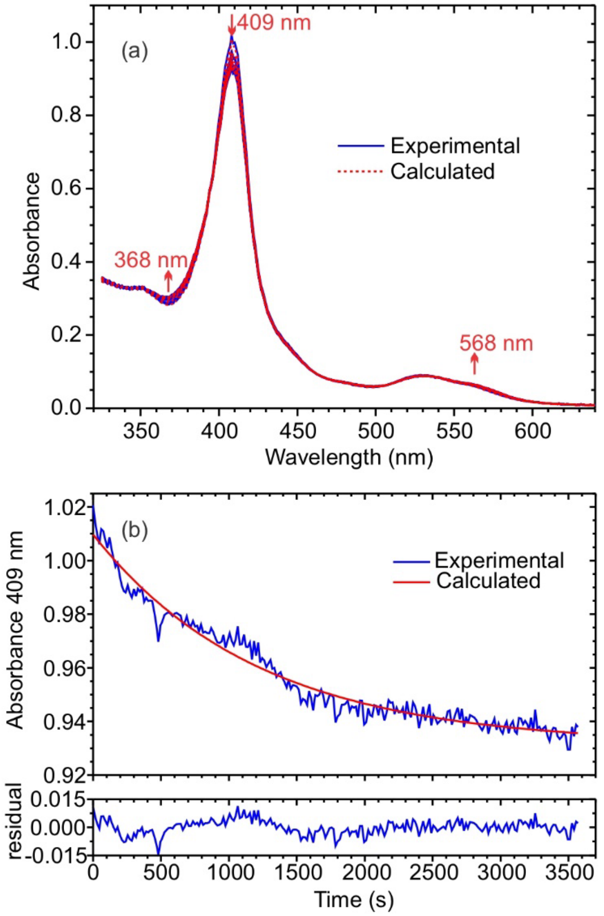

Figure S1. (a) Blue traces: UV/Vis spectral changes observed after mixing 1.6 μM R103Q with 2 mM nitrite and a 1:1 mixture of Ru^{II}:Ru^{III} (600 μM total ruthenium) in a pH 7 HEPES buffer. Dashed red traces: least-squares best fit with Eq. S1. The reaction mixture was monitored at 15s intervals, but for clarity, the selected traces are at 600 s intervals. (b) Blue trace: absorbance vs. time slice taken at the 409 nm maximum of the data set above. Red trace: least-squares best fit with Eq. S1.

The blue trace in Fig. S2 shows the spectral component $\mathbf{S_3}$ generated by fitting the SVD-processed Fig. S1 data with Eq. S1, while the dashed red trace is the least-squares best fit obtained using known extinction coefficient spectra of $R103Q_{ox}$ and nitrite. In contrast to the $\mathbf{S_0}$ component obtained when nitrite-loaded R103Q was reduced with pure Ru^{II} (main text Fig. 3), the fit of $\mathbf{S_3}$ with the $R103Q_{ox}$ and nitrite extinction coefficient spectra is excellent, showing that no appreciable heme reduction takes place in the mixing time (10 – 50 s). In the Fig. 2 experiment, pure Ru^{II} was able to rapidly reduce a 6-coordinate bis-his ligated low-spin heme, probably heme 4, which has the second-highest midpoint potential after the active site heme (about -100 mV vs SHE).[1] This in turn seemed to facilitate active site heme reduction, as evidenced by the broad absorbance band underlying the sharp 551 nm band in the $\mathbf{S_0}$ spectrum of Fig. 3.[6, 7] The 1:1 Ru^{II}:Ru^{III} mixture of the Fig. S1 experiment poises the solution at +50 mV vs SHE, and at this higher potential, heme 4 reduction is inaccessible, which in turn appears to have made fast active site heme reduction inaccessible as well.

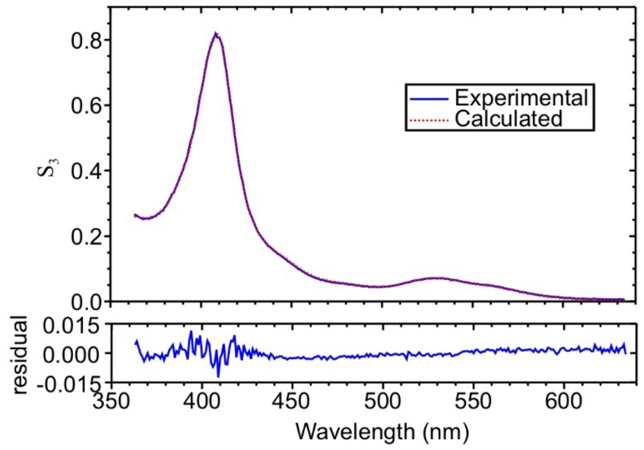

Figure S2. Blue trace: spectral component $\mathbf{S_3}$ generated by fitting the SVD-processed Fig. S1 data with Eq. S1. Dashed red trace: least-squares best fit obtained using known extinction coefficient spectra of R103Q$_{ox}$ and nitrite.

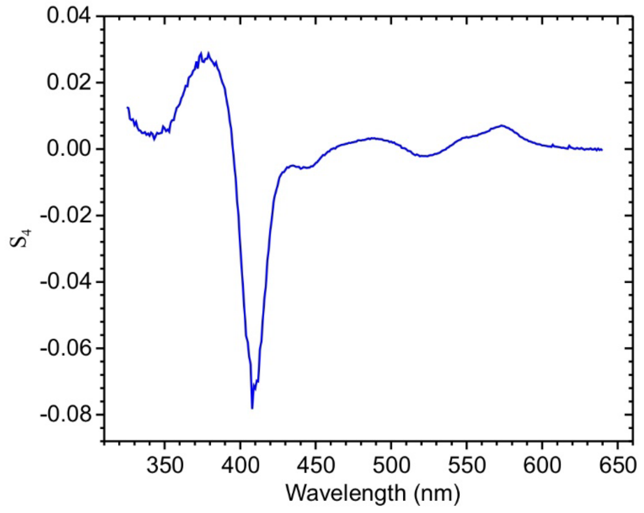

Figure S3. Spectral component $\mathbf{S_4}$ generated by fitting the SVD-processed Fig. S1 data with Eq. S1. Note the similarity to $\mathbf{S_2}$ in Fig. 4, the spectrum obtained at infinite time when nitrite-loaded R103Q was reduced with pure Ru^{II}.

Figure S3 shows the spectral component $\mathbf{S_4}$ generated by fitting the SVD-processed Fig. S1 data with Eq. S1. This component is identical in its essential features to $\mathbf{S_2}$ generated by fitting the Fig. 2 data, obtained when nitrite-loaded R103Q ccNiR was reduced with pure Ru^{II}, to Eq. 1. The only notable difference between Figs. S3 and 4b is the absence of a 552 nm feature in Fig. S3, which is consistent with there having been no initial heme 4 reduction when the enzyme is reduced in a 1:1 mixture of Ru^{II} and Ru^{III}. Furthermore, the Fig. S3 spectral component also appears to be the same or very similar to the one seen when nitrite-loaded R103Q ccNiR is exposed to the weak reductant TMPD for an hour.[8]

S6. Reactivity of nitrite-loaded Y206F with Ru^{II}

Figure S4 shows with blue traces the spectral changes observed at selected times after mixing roughly 1 µM Y206F with 2 mM nitrite and 1 mM Ru^{II}, in a pH 7 HEPES buffer. SVD analysis showed that two spectral components were needed to faithfully reconstruct a noise-reduced absorbance matrix. The SVD-treated data were then fit with the empirical 2-exponential equation S2 using a global fitting routine (red traces, Fig. S4a).[4, 5] In Eq. S2, $A_{\lambda,t}$ is the absorbance obtained at wavelength λ and time t, $\mathbf{S_5}$ is the spectral component present at t = 0, and $\mathbf{S_6}$ is a difference spectrum that represents the spectral change that would be seen after infinite time. The rate constants k_{app4}, k_{app5}, and *frac*, are adjustable parameters that are

$$\Delta A_{\lambda,t} = S_{5(\lambda)} + S_{6(\lambda)}\left\{\left[1-exp\left(-k_{app4}t\right)\right] + frac\cdot\left[exp\left(-k_{app5}t\right) - exp\left(-k_{app4}t\right)\right]\right\} \quad \text{Eq. S2}$$

manually adjusted to obtain the least-squares best fit of the experimental data (the value of *frac* is restricted to between 0 and 1). Figure S4b shows an absorbance vs. time slice from the Fig. S4a data set obtained at 409 nm, which is the Soret maximum for Y206F. The blue trace is experimentally obtained, the red one is the least-squares best fit with Eq. S2.

The blue trace in Fig. S5a shows the spectral component $\mathbf{S_5}$ that was obtained by fitting the Fig. S4a data with Eq. S2. The component $\mathbf{S_5}$ was in turn fit with the independently obtained extinction coefficient spectrum of fully oxidized Y206F (Y206F$_{ox}$) and nitrite; nitrite absorbs only slightly, and 1 mM

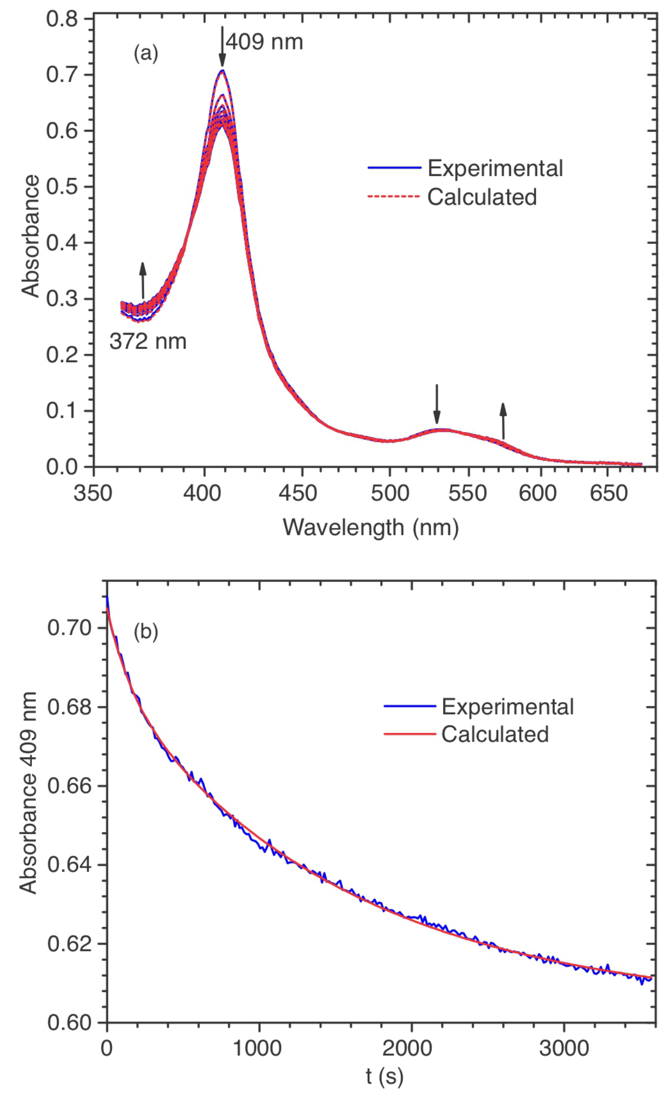

Figure S4. (a) Blue traces: UV-vis spectral changes observed after mixing ~1.5 µM Y206F with 2 mM nitrite and 1 mM Ru^{II}, in a pH 7 HEPES buffer. Dashed red traces: least-squares best fit with Eq. S2. The reaction mixture was monitored at 15s intervals, but for clarity, the selected traces are at 500 s intervals. (b) Blue trace: absorbance vs. time slice taken at the 409 nm maximum of the data set above. Red trace: least-squares best fit with Eq. S2. Best fit parameter values: k_{app4} = 0.0065 s^{-1} ($t_{0.5}$ = 110 s), k_{app5} = 0.00065 s^{-1} ($t_{0.5}$ = 1070 s), *frac* = 0191.

Ru^{II} has negligible absorbance, in this wavelength range. Note that the best fit of $\mathbf{S_5}$ in Fig. S5a with the $Y206F_{ox}$ extinction coefficient spectrum leaves a substantial residual spectrum (Fig. S5b), with absorbance increases at 387 nm, 427 nm and 563 nm, and a decrease at 407 nm. The absorbance decrease at 407 nm and increase at 427 nm is a pattern similar to the one seen in main text Fig. 3b for R103Q, and as explained in the main text, this pattern is characteristic of *c*-heme reduction.[9, 10] Thus, it appears that the Y206F variant is also reduced by Ru^{II} within the mixing time of the experiment. However, in the case of R103Q, the t=0 spectrum also shows a sharp absorbance increase at ~552 nm that is characteristic of low-spin bis-His ligated heme reduction (Fig. 3b).[9, 10] Such an increase is lacking in the Fig. S5b difference spectrum. Instead, one observes a broad absorbance increase centered at 563 nm that is more reminiscent of wild type active site heme 1 reduction to an $\{Fe_{H1}NO\}^7$ state, though slightly red-shifted from the feature seen in the earlier report.[6, 7]

Figure S6 shows the spectral component $\mathbf{S_6}$ that was obtained by fitting the Fig. S4a data to Eq. S2. $\mathbf{S_6}$ growth is empirically biphasic; the first 20% of the component grows with a half-life of ~100 s (1.6 min) and the other 80% grows with a half-life of ~1100 s (20 min). Neither half-life varies appreciably when the initial Ru^{II} concentration is varied from 100 µM – 1 mM (Fig. S7). The main features in $\mathbf{S_6}$ are the absorbance increases at 372 nm 575 nm, and the decreases at 409 nm, 442 nm, and 523 nm. These features are very similar to the ones seen within an hour of exposing nitrite-loaded R103Q to Ru^{II} (Fig. 4), except that the features associated with heme 4 re-oxidation are absent. Follow-up EPR experiments confirmed that, as in the case of R103Q, spectral component $\mathbf{S_6}$ corresponds to a 5-coordinate $\{Fe_{H1}NO\}^7$ Y206F active site moiety (main text, Fig. 7c).

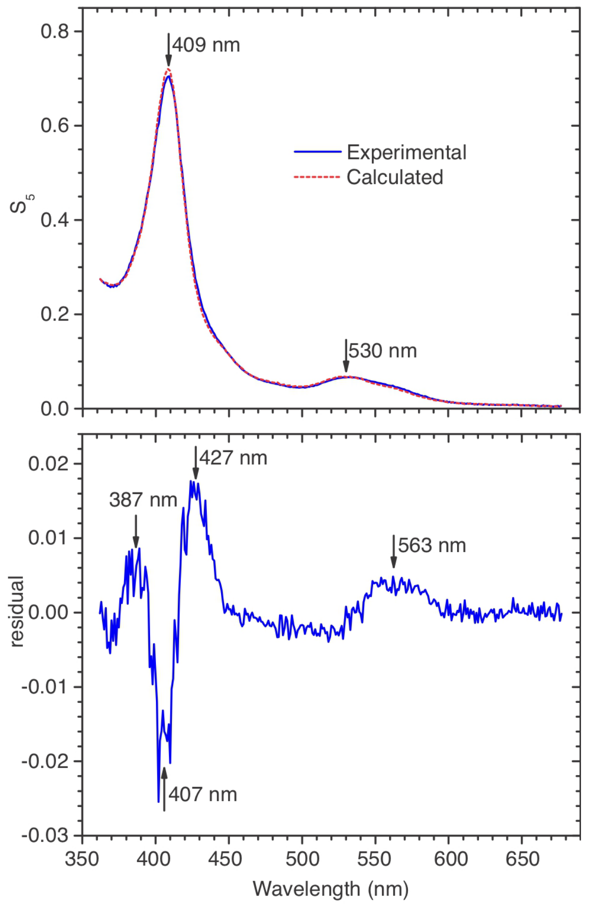

Figure S5. (a) Blue trace: spectral component $\mathbf{S_5}$ generated by fitting the SVD-processed Fig. S4a data with Eq. S2. Red trace: least-squares best fit obtained using known extinction coefficient spectra of resting (fully oxidized) Y206F and nitrite. The calculated concentration of Y206F was 1.3 µM. (b) Difference spectrum obtained by subtracting the red spectrum in Fig. S5a from the blue one.

S7. R103Q can catalyze nitrite reduction to NO· by TMPD and hexaammineruthenium(II)

CcNiR$_{WT}$ catalyzes reduction of nitrite to NO· by weak reductants such as ferricyanide or *N,N,N′,N′*-tetramethyl-*p*-phenylenediamine (TMPD).[7] The following experiments show that the R103Q variant similarly catalyzes reduction of nitrite to NO· by the weak reductants TMPD and hexaammineruthenium(II).

Nitric oxide formation was monitored in the presence of catalase (Cat), which rapidly and

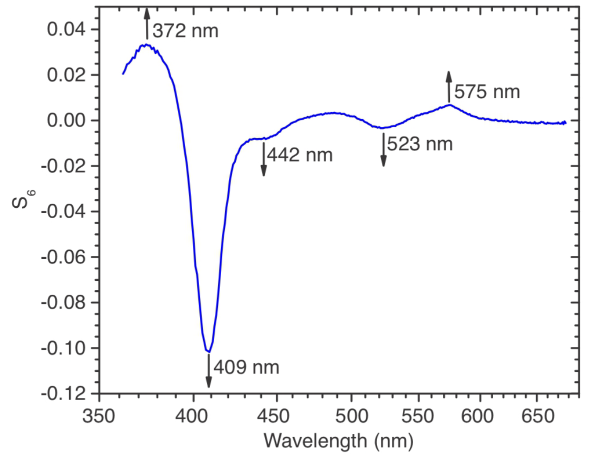

Figure S6. Spectral component $\mathbf{S_6}$ obtained by fitting the Fig. S4a data to Eq. S2.

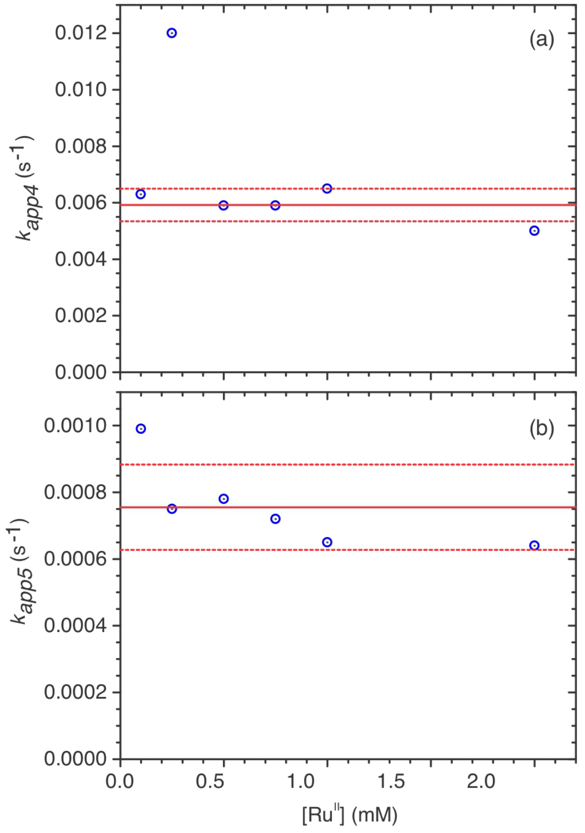

Figure S7. Dependence on [Ru^{II}] of the (a) k_{app4} and (b) k_{app5} values obtained from fitting data like those of Fig. S4a to Eq. S2. In all cases, [NO_2^-] = 2 mM and [Y206F] ~1.5 µM. Average k_{app} values: k_{app4} = (5.9 ± 0.6)×10^{-3} s^{-1} (excluding the outlier at 250 µM); k_{app5} = (7.6 ± 1.3)×10^{-4} s^{-1}.

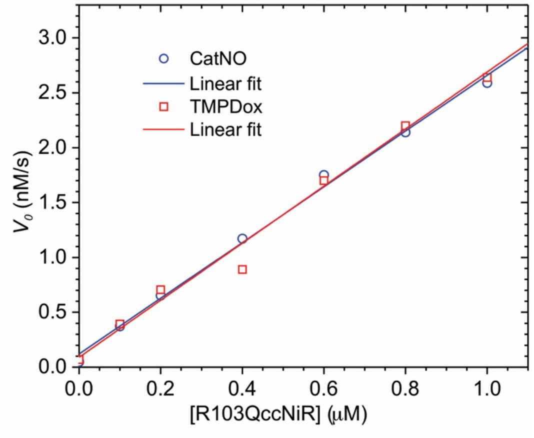

Figure S8. Blue circles: initial rate of CatNO formation vs R103Q concentration when TMPD is the electron source; red squares: initial rate of $TMPD_{ox}$ formation vs R103Q concentration. The blue and red lines are the linear least-squares best fits to each data set. The data sets were collected at pH 7.0, with a nitrite concentration of 2 mM, a TMPD concentration of 1mM and a catalase concentration of 4 µM. Blue line: slope = $(2.54\pm0.07)\times10^{-3}$ s^{-1}, intercept = $(1.2\pm0.4)\times10^{-10}$ M/s; red line: $(2.6\pm0.1)\times10^{-3}$ s^{-1},

$$\text{TMPD} + NO_2^- + 2H^+ \rightleftharpoons \text{TMPD}_{ox} + \text{NO}\cdot + H_2O$$

$$\text{NO}\cdot + \text{Cat} \rightarrow \text{CatNO}$$

Scheme S3. Trapping of NO· by catalase. Catalase (Cat) and the nitrosylated product (CatNO) have very different colors. The appearance of $TMPD_{ox}$ can be simultaneously determined from its distinct blue color.

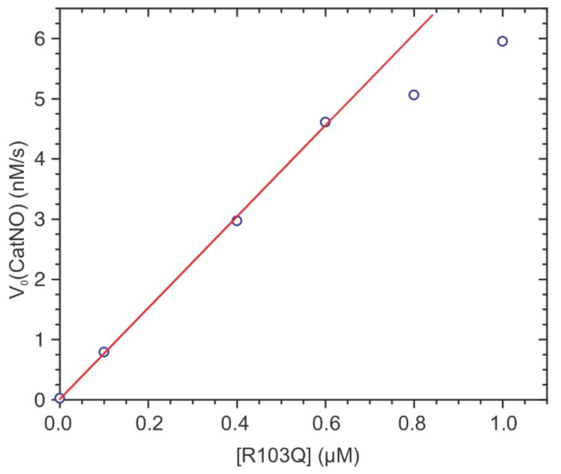

Figure S9. Blue circles: initial rate of CatNO formation vs R103Q concentration when Ru^{II} is the electron source; The red line is the linear least-squares best fit to the first four points of the data set (slope = $(7.6\pm0.1)\times10^{-3}$ s^{-1}). The data set was collected at pH 7.0, with a nitrite concentration of 2 mM, a Ru^{II} concentration of 400 µM and a catalase concentration 4 µM.

strongly binds NO· to form nitrosylated catalase (CatNO) in a process accompanied by a distinct color change (step 2, Scheme S3). In the case where TMPD is the electron source, the electron transfer rate (step 1, Scheme S3) could be obtained simultaneously with the NO· formation rate by monitoring the appearance of oxidized TMPD ($TMPD_{ox}$), which has a distinct blue color. The assay procedures are described in detail in ref. 7. Figure S8 summarizes the results of a series of experiments in which solutions initially containing 2 mM nitrite, 1mM TMPD, 4 µM catalase, and varying concentrations of R103Q, in 50 mM HEPES pH 7 buffer, were allowed to react for 1h. The blue circles and red squares in Fig. S8 show how the initial rates of CatNO and $TMPD_{ox}$ formation, respectively, vary with R103Q concentrations. Both the V_0(CatNO) and V_0($TMPD_{ox}$) vs [R103Q] data sets were well fit with nearly overlapping straight lines, which is consistent with the conjecture that virtually all TMPD oxidation was associated with reduction of nitrite to NO·, as shown in step 1 of Scheme S3. The slope of the V_0(CatNO) vs [R103Q] line (blue) is $(2.54\pm0.07)\times10^{-3}$ s^{-1}, while that of the V_0($TMPD_{ox}$) vs [R103Q] line (red) is $(2.6\pm0.1)\times10^{-3}$ s^{-1}, identical within experimental error. The y-intercepts of the V_0(CatNO) and V_0($TMPD_{ox}$) vs [R103Q] lines, $(1.2\pm0.4)\times10^{-10}$ M/s and $(9\pm7)\times10^{-11}$ M/s, respectively, are close to zero, showing that little TMPD oxidation or NO· formation take place in the absence of R103Q.

The NO· trapping experiment repeated using Ru^{II} as the electron source yielded similar results (Fig. S9). Figure S9 shows that the rate of CatNO formation was linearly dependent on [R103Q], at least up to 0.6 µM R103Q. The slope of the line is $(7.6\pm0.1)\times10^{-3}$ s^{-1}, which is about 3× higher than observed when TMPD is the reductant ($(2.54\pm0.07)\times10^{-3}$ s^{-1}, Fig. S8). Thus, the rate of NO· formation is modestly

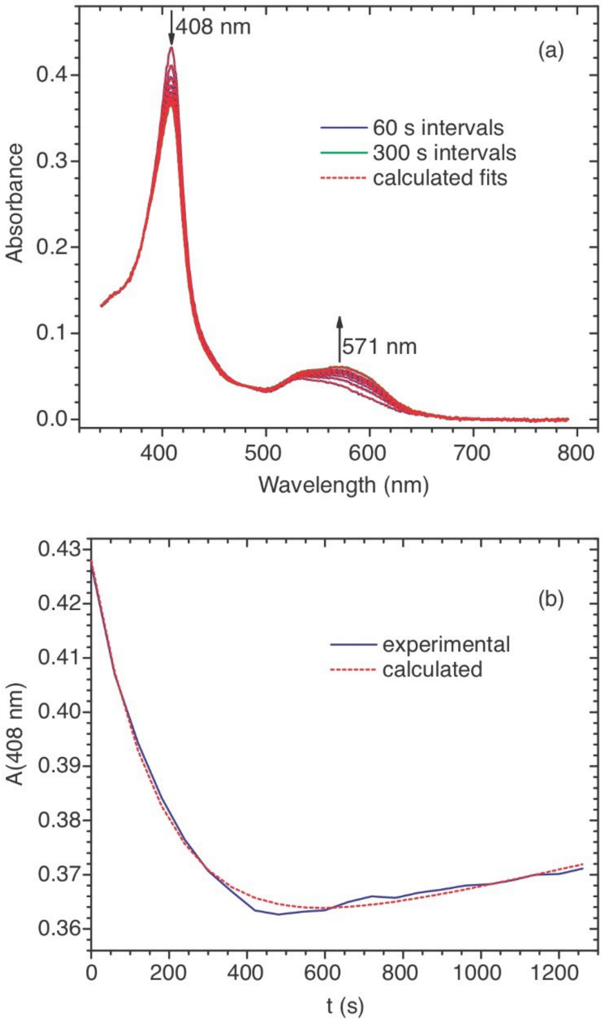

Figure S10. (a) Blue and green traces: UV/Vis spectral changes observed in a 1 mm pathlength cuvette containing 10 μM R103Q, 2 mM nitrite, and 20 μM reduced I4S, in the 30 minutes after mixing the reagents (pH 7 HEPES buffer). Dashed red traces: least-squares best fit with Eq. S3. (b) Blue trace: absorbance vs. time slice taken at the 408 nm maximum of the data set above. Dashed red trace: least-squares best fit with Eq. S3. The least-squares best fit to the data was obtained with k_{app6} = 0.0055 s^{-1} ($t_{0.5}$ = 126 s).

higher when the stronger reductant is the electron source. In Fig. S9, the intercept is effectively zero ($(1\pm5)\times10^{-11}$ nM/s). Beyond 0.6 μM R103Q, the dependence appears to deviate from linearity; however, this is probably an artifact. It appears that, at high R103Q concentrations, the rate of CatNO formation when pure Ru^{II} is the reductant becomes high enough that significant CatNO is formed during the mixing time, and consequently one is no longer measuring the initial rate of CatNO formation in these experiments.

S8. Reduction of nitrite-loaded R103Q with reduced indigo tetrasulfonate for EPR analysis

As shown in section S7, R103Q catalyzes reduction of nitrite to NO· by weak reductants, and as mentioned in the main text, this can limit the accumulation of reduced R103Q intermediates when the ratio R103Q:reductant is high. This section describes in detail the procedure used to maximize accumulation for EPR studies of the reduced R103Q species with the Fig. 4 spectral characteristics (blue trace, main text Fig. 4) when using $I4S_{red}$ as the electron source.

The blue and green traces in Fig. S10a show the spectral changes observed in a solution initially containing 10 μM R103Q, 2 mM nitrite, and 20 μM reduced I4S, in the 30 minutes after mixing the reagents. The cuvette pathlength for this experiment was 0.1 cm to accommodate the more concentrated solution. SVD analysis showed that three spectral components were needed to faithfully reconstruct a noise-reduced absorbance matrix. The red dashed traces are the least-squares best fits of the SVD-treated data to Eq. S3, where $A_{\lambda,t}$ is the absorbance obtained at wavelength λ and time t, $\mathbf{S_7}$ is the spectral component present

$$A_{\lambda,t} = S_{7(\lambda)} + S_{8(\lambda)}\left[1-\exp(-k_{app6}t)\right] + S_{9(\lambda)}t \qquad \text{Eq. S3}$$

at t = 0, $\mathbf{S_8}$ is a spectral component that grows in exponentially at a rate governed by k_{app6}, and $\mathbf{S_9}$ is a spectral component that changes linearly with time. Figure S10b shows an absorbance vs. time slice from the Fig. S10a data set, obtained at 408 nm. The slice shows that the linear change in absorbance at

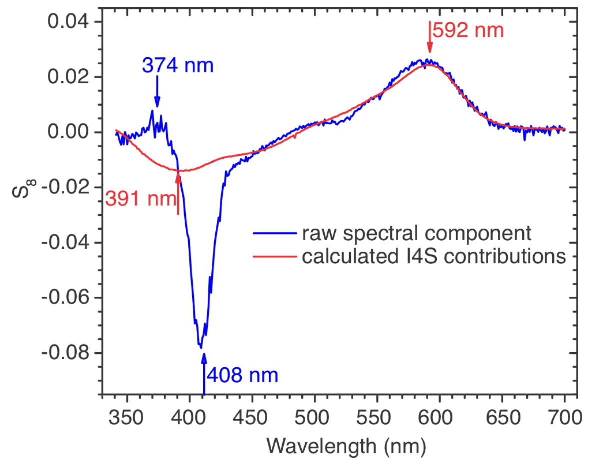

Figure S11. Blue trace: spectral component $\mathbf{S_8}$ obtained from the least-squares best fit of the Fig. S10 data with Eq. 3. Dashed red trace shows the estimated contributions to the component from $I4S_{ox}$ and $I4S_{red}$, obtained from the independently known extinction coefficient spectra of the I4S species. The blue trace in main text Fig. 6 was obtained by subtracting the estimated I4S contributions from the $\mathbf{S_8}$ component.

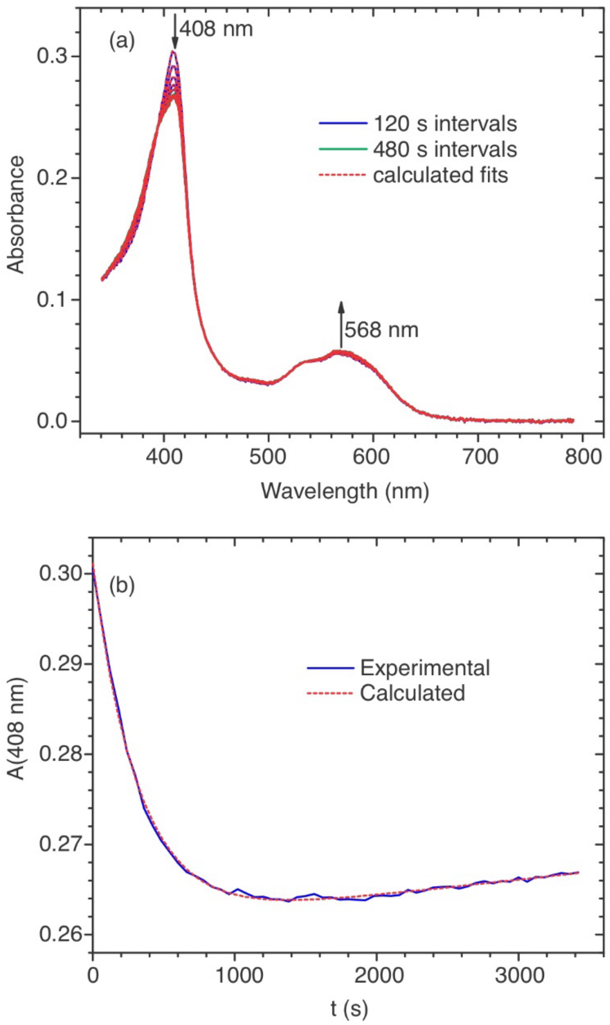

Figure S12. (a) Blue and green traces: UV/Vis spectral changes observed in the solution from Fig. S10 after adding 300 μM of the nitric oxide generator DEANO at the 30-minute mark. Dashed red traces: least-squares best fit with Eq. S4. (b) Blue trace: absorbance vs. time slice taken at the 408 nm maximum of the data set above; the moment of DEANO addition is taken as t = 0. Dashed red trace: least-squares best fit with Eq. S4. The least-squares best fit to the data was obtained with k_{app7} = 0.0031 s^{-1} ($t_{0.5}$ = 224 s).

408 nm goes in the opposite direction to the initial exponential change. The blue trace in Fig. S11 shows the spectral component $\mathbf{S_8}$ generated by fitting the SVD-processed Fig. S10 data with Eq. S3, while the dashed red trace shows the estimated contributions to the component from $I4S_{ox}$ and $I4S_{red}$, obtained from the independently known extinction coefficient spectra of the I4S species. The blue trace in main text Fig. 6 was obtained by subtracting the estimated I4S contributions from the $\mathbf{S_8}$ component.

After 30 minutes, an aliquot of DEANO was added to the Fig. S10 solution, diluting it to 88% of its original concentration; the DEANO concentration in the mixture after the addition was 300 μM. The blue and green traces in Fig. S12a show the spectral changes observed in the hour following the DEANO addition. SVD analysis showed that three spectral components were needed to faithfully reconstruct a noise-reduced absorbance matrix, and the data were fit with Eq. S4, which is analogous to Eq. S3 (red dashed traces, Fig. S12a). Figure S12b shows an absorbance vs. time slice from

$$A_{\lambda,t} = S_{10(\lambda)} + S_{11(\lambda)}\left[1 - \exp(-k_{app7}t)\right] + S_{12(\lambda)}t \quad \text{Eq. S4}$$

the Fig. S12a data set, obtained at 408 nm. The spectral changes seen in Fig. S12 are similar in the Soret region to those seen in Fig. S10; however, the changes seen above 500 nm are noticeably smaller in Fig. S12 than in Fig. S10. The exponentially growing spectral component $\mathbf{S_{11}}$ (Fig. S13) shows the significance

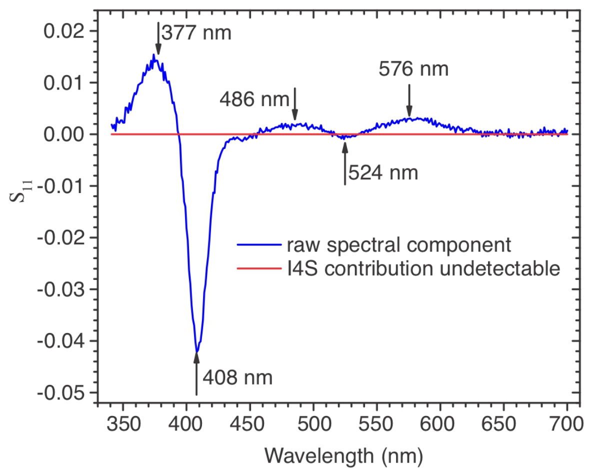

Figure S13. Blue trace: spectral component $\mathbf{S_{11}}$ obtained from the least-squares best fit of the Fig. S12 data to Eq. 4. There were no detectable contributions to $\mathbf{S_{11}}$ from $I4S_{ox}$ and $I4S_{red}$. The red trace in main text Fig. 5 was obtained by adding the $\mathbf{S_{11}}$ component to the portion of the $\mathbf{S_8}$ component attributable solely to R103Q.

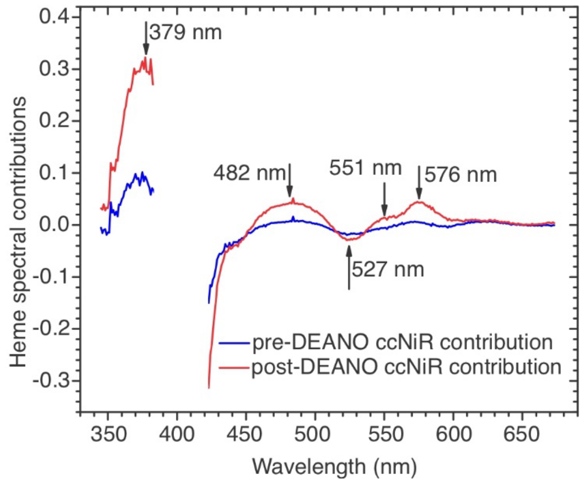

Figure S14. Blue trace: heme contribution to the UV/Vis spectral component that grows exponentially in a reaction mixture initially containing 60 μM R103Q, 2 mM nitrite, and 120 μM $I4S_{red}$. Red trace: heme contribution to the component that grows exponentially after adding 30 equivalents of DEANO to the reaction mixture at the 30-minute mark. The pathlength of the cuvette containing the reaction mixture was 1 mm; off-scale data between 384 nm and 422 nm were deleted prior to analysis.

of this observation. In contrast to component $\mathbf{S_8}$, $\mathbf{S_{11}}$ has no detectable contribution from I4S oxidation and can be attributed exclusively to changes in the R103Q heme spectra. The red trace in main text Fig. 6 is the sum of the $\mathbf{S_8}$ and $\mathbf{S_{11}}$ components. It has essentially the same shape as the blue trace from component $\mathbf{S_8}$ but a significantly greater amplitude, demonstrating that addition of NO· (from DEANO) to the R103Q solution increases the concentration of the species that grows in when nitrite-loaded R103Q is exposed to two equivalents of $I4S_{red}$ for tens of minutes.

EPR experiments required solutions containing at least 60 μM R103Q and 120 μM $I4S_{red}$. At such high concentrations, the region of the UV/Vis spectra between 384 nm and 422 nm is off scale. However, as shown in Fig. S14, spectral features from parts of the UV/Vis spectrum that aren't off scale at the high reagent concentrations confirm that the procedure used to reduce 10 μM R103Q is scalable to the higher concentrations. The Figure S14 spectral components were obtained from a solution in which 60 μM of R103Q was allowed to react with 2 mM nitrite and 120 μM $I4S_{red}$ for 30 minutes, at which point 30 equivalents of DEANO were added and reaction progress was followed for an additional 60 minutes. After deleting the off-scale data points between 384 nm and 422 nm, SVD analysis of the pre- and post-DEANO addition spectra showed that three spectral components could still faithfully reconstruct the corresponding noise-reduced absorbance matrices, and that the corresponding data could be fit with equations analogous to Eqs. S3 and S4. Figure S14 shows the heme contributions to the spectral components that correspond to the $\mathbf{S_8}$ and $\mathbf{S_{11}}$ components obtained in the fits of the 10 μM R103Q data (main text, Fig. 6). Note the similarities between the components of Figs. 6 and S14. In particular, the absorbance maximum seen at ~379 nm is characteristic of the species that appears tens of minutes after exposing nitrite-loaded R103Q (and the Y206F and H257Q ccNiR variants) to weak reducing agents such as TMPD, Ru^{II}, and $I4S_{red}$. The same feature is not observed after reducing nitrite-loaded wild type ccNiR under comparable conditions.[6, 7]

S9. Additional EPR data and analyses

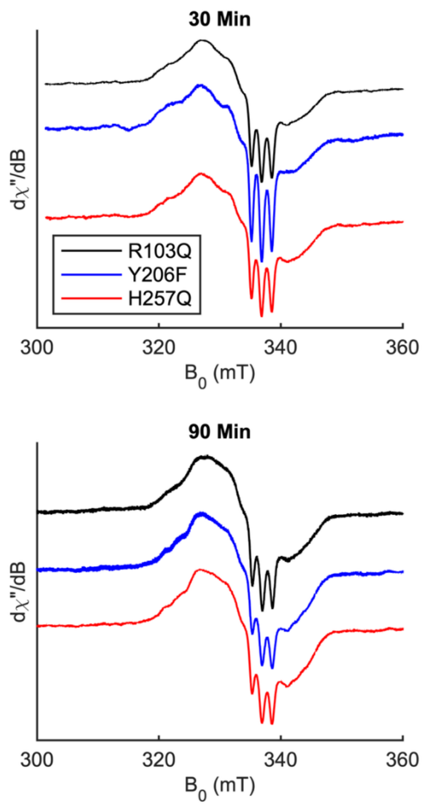

Figure S15: EPR spectra obtained at 100 K for ~60 µM CcNiR (R103Q- Black, Y206F- Blue, H257Q- Red), 2 mM nitrite, and 120 µM $I4S_{red}$ after incubating for 30 min at room temperature (Top Panel) and after addition of 30 equivalents of the NO· generating species DEANO (Bottom Panel) and incubating for 90 min at room temperature.

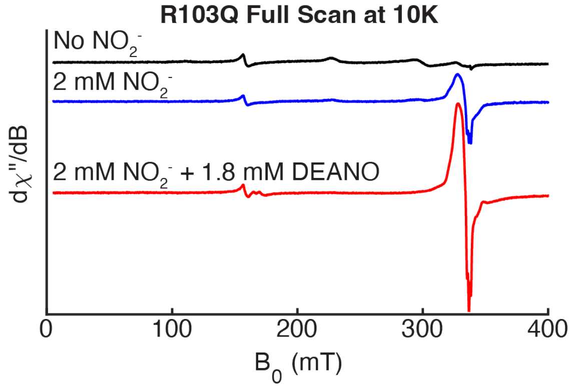

Figure S16. Full scale EPR spectrum obtained at 10 K for a solution initially containing 60 µM R103Q, 2 mM nitrite, and 120 µM $I4S_{red}$ before (blue trace) and after (red trace) addition of 30 equivalents of the NO· generating species DEANO. The spectrum of the enzyme in the absence of nitrite (black trace) is provided for comparison. The spectra shown in Fig. 7a of the main text expand the region between 300 mT and 360 mT of the full-scale spectra shown here.

Table S3. Parameters used to simulate the reduced variant EPR spectra

	g_1	g_2	g_3	A_1	A_2	A_3	H_1	H_2	H_3	Comp/Total
**5- Coordinate A**	2.1057	2.0245	2.0102	45	55	48.5	62.1992	61.4763	21.8738	26%
**5- Coordinate B**	2.06	2.058	2.0125	44	57	50	252.35	53.1058	35.2369	37%
**6- Coordinate**	2.097	2.018	1.985	62	50	40	60	60	80	37%
**SysA[11]**	2.1057	2.0245	2.0102	45	55	48.5	62.1992	61.4763	21.8738	
**SysB[9]**	2.06	2.058	2.0125	44	57	50	252.35	53.1058	35.2369	

Anisotropic strain (H) in MHz and Hyperfine coupling (A) in MHz

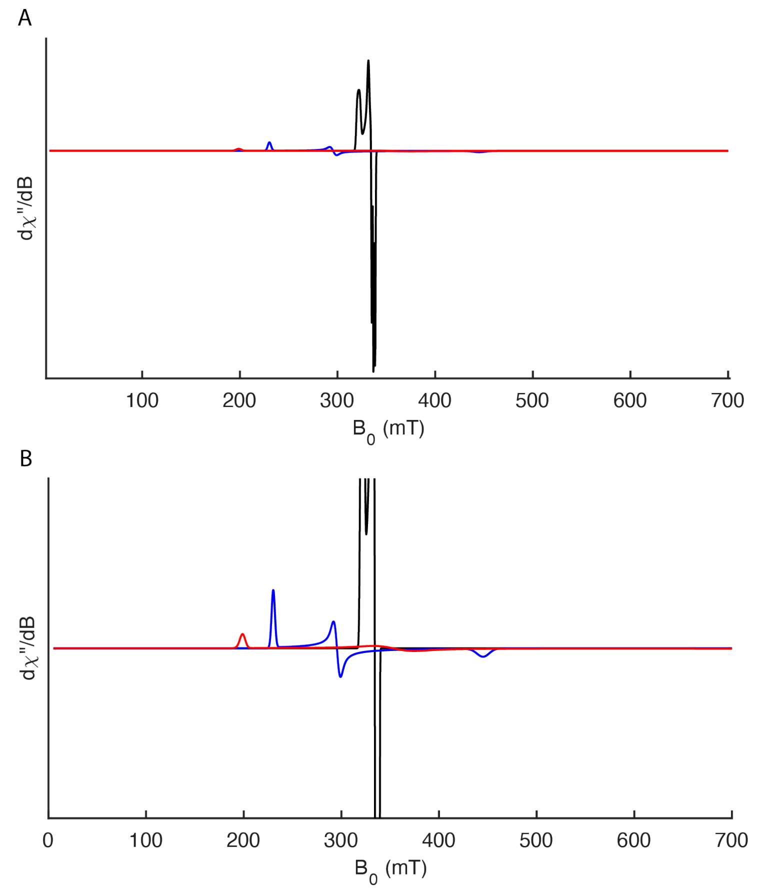

Figure S17. Simulated EPR Intensities of CcNiR. Simulated are a five-coordinate $\{FeNO\}^7$ in Black (species A in Table S3), one bis-His heme with *g*-values of (2.94, 2.29, 1.52) and g-strain of (0.05, 0.05, 0.05) in Blue,[1] and a second bis-His ligated heme with *g*-values of (3.39, 1.9, 0.95) and g-strain of (0.2, 0.2, 0.1) in Red. These values were simulated to represent a typical Rhombic and High Axial Low Spin (HALS) species. Equal weight was applied to all simulations to highlight the expected intensity differences observed in the experimental spectra. Notice that the features from species A have a much greater amplitude than those associated with the bis-His ligated hemes, because the $\{FeNO\}^7$ spectrum is so much narrower. This explains why the Fig. S16 features seen in the absence of NO_2^- are so much weaker than those associated with the 5-coordinate $\{Fe_{H1}NO\}^7$ moiety.

S10. References

1. Stein, N.; Love, D.; Judd, E. T.; Elliott, S. J.; Bennett, B.; Pacheco, A. A., Correlations between the electronic properties of *Shewanella oneidensis* cytochrome *c* nitrite reductase (ccNiR) and its structure: effects of heme oxidation state and active site ligation. *Biochemistry* **2015,** *54*, 3749-3758.
2. Youngblut, M.; Judd, E. T.; Srajer, V.; Sayyed, B.; Goelzer, T.; Elliott, S. J.; Schmidt, M.; Pacheco, A. A., Laue crystal structure of *Shewanella oneidensis* cytochrome *c* nitrite reductase from a high-yield expression system. *J. Biol. Inorg. Chem.* **2012,** *17,* 647-662.
3. Judd, E. T.; Stein, N.; Pacheco, A. A.; Elliott, S. J., Hydrogen bonding networks tune proton-coupled redox steps during the enzymatic six-electron conversion of nitrite to ammonia. *Biochemistry* **2014,** *53*, 5638-5646.
4. Koebke, K. J.; Pauly, D. J.; Lerner, L.; Liu, X.; Pacheco, A. A., Does the oxidation of nitric oxide by oxymyoglobin share an intermediate with the metmyoglobin-catalyzed isomerization of peroxynitrite? *Inorg. Chem.* **2013,** *52*, 7623-7632.
5. Koebke, K. J.; Waletzko, M. T.; Pacheco, A. A., Direct monitoring of the reaction between photochemically generated nitric oxide and *Mycobacterium tuberculosis* tuncated hemoglobin N wild type and variant forms: an assessment of computational mechanistic predictions. *Biochemistry* **2016,** *55*, 686-696.
6. Ali, M.; Stein, N.; Mao, Y.; Shahid, S.; Schmidt, M.; Bennett, B.; Pacheco, A. A., Trapping of a putative intermediate in the cytochrome *c* nitrite reductase (ccNiR)-catalyzed reduction of nitrite: implications for the ccNiR reaction mechanism *J. Am. Chem. Soc.* **2019,** *141*, 13358-13371.
7. Shahid, S.; Ali, M.; Legaspi-Humiston, D.; Wilcoxen, J.; Pacheco, A. A., A kinetic investigation of the early steps in cytochrome *c* nitrite reductase (ccNiR)-catalyzed reduction of nitrite. *Biochemistry* **2021,** *60*, 2098-2115.
8. Alam, S. The effects of an R103Q mutation on the chemical and physical properties of the enzyme cytochrome *c* nitrite reductase (ccNiR). University of Wisconsin-Milwaukee, 2022.
9. Arciero, D. M.; Collins, M. J.; Haladjian, J.; Bianco, P.; Hooper, A. B., Resolution of the 4 Hemes of Cytochrome-C554 from Nitrosomonas-Europaea by Redox Potentiometry and Optical Spectroscopy. *Biochemistry* **1991,** *30* (48), 11459-11465.
10. Marritt, S. J.; Kemp, G. L.; Xiaoe, L.; Durrant, J. R.; Cheesman, M. R.; Butt, J. N., Spectroelectrochemical characterization of a pentaheme cytochrome in solution and as electrocatalytically active films on nanocrystalline metal-oxide electrodes. *J. Am. Chem. Soc.* **2008,** *130*, 8588-8589.
11. Gunn, A.; Derbyshire, E. R.; Marletta, M. A.; Britt, R. D., Conformationally distinct five-coordinate heme-NO complexes of soluble guanylate cyclase elucidated by multifrequency electron paramagnetic resonance (EPR). *Biochemistry* **2012,** *51*, 8384-8390.
